# Does that sound right? A novel method of evaluating models of reading aloud: Rating nonword pronunciations

**DOI:** 10.3758/s13428-022-01794-8

**Published:** 2022-06-01

**Authors:** Michele Gubian, Ryan Blything, Colin J. Davis, Jeffrey S. Bowers

**Affiliations:** 1 Institute of Phonetics and Speech Processing, LMU Munich, Munich, Germany; 2 School of Psychology, Aston University, Aston, UK; 3 School of Psychological Science, University of Bristol, Bristol, UK

**Keywords:** Reading aloud, Computational reading models, Generalization, Pronunciation

## Abstract

Nonword pronunciation is a critical challenge for models of reading aloud but little attention has been given to identifying the best method for assessing model predictions. The most typical approach involves comparing the model’s pronunciations of nonwords to pronunciations of the same nonwords by human participants and deeming the model’s output correct if it matches with any transcription of the human pronunciations. The present paper introduces a new ratings-based method, in which participants are shown printed nonwords and asked to rate the plausibility of the provided pronunciations, generated here by a speech synthesiser. We demonstrate this method with reference to a previously published database of 915 disyllabic nonwords (Mousikou et al., 2017). We evaluated two well-known psychological models, RC00 and CDP++, as well as an additional grapheme-to-phoneme algorithm known as Sequitur, and compared our model assessment with the corpus-based method adopted by Mousikou et al. We find that the ratings method: a) is much easier to implement than a corpus-based method, b) has a high hit rate and low false-alarm rate in assessing nonword reading accuracy, and c) provided a similar outcome as the corpus-based method in its assessment of RC00 and CDP++. However, the two methods differed in their evaluation of Sequitur, which performed much better under the ratings method. Indeed, our evaluation of Sequitur revealed that the corpus-based method introduced a number of false positives and more often, false negatives. Implications of these findings are discussed.

**Supplementary Information:**

The online version contains supplementary material available at 10.3758/s13428-022-01794-8.

## Introduction

Experimental psychologists have studied the processes underlying reading aloud for well over a century (e.g. Huey, 1908). According to a longstanding account, skilled readers make use of two distinct procedures operating in parallel to translate orthography into phonology: a lexical procedure that looks up the pronunciation of familiar words, and a rule-based procedure that computes a pronunciation via the application of grapheme-phoneme correspondence rules such as ea→ /i:/ (e.g. Coltheart, 1987; Morton, 1980; Ellis & Young, 2013). This dual-route account was subsequently implemented as a computational model, the dual-route cascaded model (DRC; Coltheart et al.,, 2001). A key motivation for positing two distinct procedures is to explain the ability to read aloud both irregular words like “pint” and nonwords like “slint”. However, this dual-route account was challenged by Seidenberg and McClelland (1989), who argued that a gradient descent learning mechanism could give rise to a set of probabilistic associations that are sufficient to explain how both irregular words and nonwords can be read aloud. Computational models instantiating this theoretical claim were introduced by Seidenberg and McClelland (1989) and Plaut et al., (1996). A third approach is to combine aspects of the above two accounts by combining a lexical procedure with a probabilistic association pathway that learns to map graphemes to phonemes (Perry et al., 2007, 2010).

### Assessing models of reading aloud: Rule-based, analogy-based and corpus-based methods of scoring nonword pronunciation

These models of reading aloud can be tested by examining their ability to generalise their knowledge of spelling-sound correspondences to novel stimuli, i.e. their ability to name nonwords. Indeed, a fundamental challenge to Seidenberg and McClelland (1989)’s model was offered by Besner et al., (1990), whose analysis of the model’s nonword naming performance concluded that the model “failed to produce the correct phonological response to almost 50% of stimuli” (p. 434). Plaut et al., (1996) conceded that the model’s ability to name nonwords was significantly worse than skilled readers, but also noted that Besner et al., (1990)’s method of assessing the model was somewhat problematic. Besner et al., (1990) scored as correct any pronunciation of a nonword that followed grapheme-phoneme correspondence rules (i.e. correctness was based solely on regularity). However, this is an unfair test, as readers do not always follow grapheme-phoneme correspondence rules when pronouncing nonwords (e.g. Andrews and Scarratt, 1998; Glushko, 1979). By the same token, Coltheart et al., (2001)’s scoring of the DRC model’s naming of nonwords – which adopted the same rule-based method as Besner et al., (1990) – was overpermissive to a model that relies almost exclusively on grapheme-phoneme correspondence rules to name nonwords.

Because human readers sometimes produce nonword pronunciations that appear to be derived by analogy with real words rather than by following rules, Seidenberg et al., (1994) proposed an alternative analogy-based scoring method, according to which nonword pronunciations were scored as correct “if we could identify a plausible basis for them (either a rule or an analogy to a neighbouring word)” (p.1185). For example, their model was scored correct for pronouncing “jook” as /juk/ by analogy with the rhyming words “book”, “cook”, etc., whereas the scoring method used by Besner et al., (1990) and Coltheart et al., (2001) would score this pronunciation as incorrect (grapheme-phoneme correspondence rules imply a pronunciation rhyming with “spook”). The model scored extremely well when this method was used; for instance, its performance on the Glushko (1979) set was scored as 96.5% correct (the original participants scored 94.9% on average, according to this criterion). The same scoring method was used by Perry et al., (2007) in their assessment of their CDP+ model. They reported an error rate of 6.3% for a set of 592 monosyllabic nonwords, comparable to the error rate of 7.3% for human participants.

However, just as Coltheart et al., (2001)’s rule-based scoring of the DRC model’s nonword naming is overly generous in its assessment of that model’s performance, the analogy-based method used by Seidenberg et al., (1994) and Perry et al., (2007) to score their models seems far too lenient. For example, the nonword “jinth” *could* be pronounced with a long vowel, by analogy with the word “ninth”, but Andrews and Scarratt (1998) found that not one of their 44 participants produced this pronunciation (all participants gave the regular pronunciation that rhymes with the word “plinth”). Indeed, Andrews and Scarratt (1998) found that the nonwords with irregular body neighbours that they tested were given regular pronunciations by their participants 85% of the time, overall. Thus, although researchers may be able to point to analogies that support the irregular pronunciations produced by models, it is not clear how relevant this is to comparing models with human performance. In the same way, Pritchard et al., (2012) criticised the analogy-based scoring method as too lenient because it includes “too many pronunciation possibilities that readers simply do not consider” (p.1277). An additional limitation concerns the potential subjectivity of this measure, since a researcher must decide the extent to which position-specific or position-independent rules are considered (extreme cases of the latter may lead to particularly lenient pronunciations such as the famous “fish” pronunciation of the nonword “ghoti”).

An apparently straightforward way to overcome the problems associated with the rule-based and analogy-based scoring methods is to consider what pronunciations readers actually assign to nonwords. As Plaut et al., (1996) argued, the important question is not whether model’s pronunciations are “correct” (in the sense of following grapheme-phoneme correspondence rules), but whether these pronunciations are similar to those produced by human readers. However, assessing models on the basis of human pronunciations is not quite as straightforward as one might initially expect. One issue is that many of the older data sets in the literature do not record what pronunciations participants gave – the coding is limited to whether the pronunciation was regular (e.g. Andrews and Scarratt, 1998; Glushko, 1979).^1^

The more fundamental issue, though, is that there is considerable variability in human nonword naming (e.g. Pritchard et al.,, 2012; Mousikou et al.,, 2017). This variability challenges the notion that there is a correct pronunciation of nonwords (which in itself is another reason that the rule-based scoring method is problematic). Accommodating this pronunciation variability suggests the need for datasets (corpora) that record all of the responses given by participants and scoring methods that are appropriately sensitive to this variability.

To this end, Pritchard et al., (2012) assembled a corpus of naming responses for 412 monosyllabic nonwords (each of which was read aloud by 45 adults). All responses were transcribed and the resulting corpus was used to assess the DRC, CDP+ and CDP++ (Perry et al., 2010) models. A model’s pronunciation of each nonword was deemed correct if it matched with any of the pronunciations in the corpus. This corpus-based approach captures the variability of human responses (contrary to the rule-based scoring method used by Coltheart et al.,, 2001) but is not as lenient as the analogy-based scoring method used by Seidenberg et al., (1994) and Perry et al., (2007). Pritchard et al., (2012) found that error rates were 1.5% for DRC, 49.0% for CDP+, and 26.9% for CDP++. That is, for almost half of the nonwords the CDP+ model produced a response that was not produced by any of the 45 human readers. These results appear to pose a strong challenge to both the CDP+ and CDP++ models. However, it is important to note that the assessment of the DRC model depends on the details of the scoring method. Although it was rare for the model’s pronunciation not to correspond to one that was given by at least one participant (reflecting the frequency with which regular pronunciations are produced), for over a quarter of the nonwords the most frequent response given by participants did not correspond to the pronunciation output by DRC.

More recently, Mousikou et al., (2017) have extended the corpus-based method to disyllabic nonwords. Disyllabic words and nonwords pose additional challenges given that they contain a higher proportion of inconsistent grapheme-phoneme correspondences and raise the problem of stress assignment. Mousikou et al., (2017) constructed a corpus of disyllabic nonword naming by transcribing the responses of 41 adult participants who read aloud 915 disyllabic nonwords. This corpus demonstrates the striking variability of human responses; on average there were 5.9 alternative pronunciations per nonword, with a range from 1 to 22.

Mousikou et al., (2017) used this corpus to assess two models that are able to read aloud disyllabic nonwords, CDP++ and RC00 (Perry et al., 2010; Rastle and Coltheart, 2000). They found that the two models had different strengths, with the CDP++ model doing better at stress assignment and the Rastle and Coltheart (2000) model (abbreviated RC00) doing better at pronunciation. But the performance of both models differed from human performance: around one in four of CDP++ pronunciations and around one in eight of RC00 pronunciations were not produced by any human reader.

### Limitations of the corpus-based method

The corpora collected by Pritchard et al., (2012) and Mousikou et al., (2017) are a valuable resource for understanding human nonword naming and testing models. Nevertheless, there are some limitations of the corpus-based approach to assessing models. Some of these limitations are related to the extremely resource-intensive nature of the corpus-based approach. In the case of Mousikou et al., (2017), a single listener was asked to transcribe all the pronunciations of the 41 participants (over 37K sound recordings) into English phonemes and then compare these transcriptions to the phonemic outputs of the models. Quite apart from the Herculean nature of this task, placing such demands on a single listener raises the risk of both random coding errors and systematic biases. Indeed, recent evidence suggests that such coding errors may be quite likely, with De Simone et al., (2021) reporting that two trained transcribers of English were in only moderate agreement (*k*= .57) when transcribing a set of English pseudowords in their Experiment 1. Future model evaluation will require researchers to expand the set of nonwords used to analyse models; reliance on researchers to construct large corpora may impede progress.

Other limitations of the corpus-based method are related to the resulting data. It makes sense to compare model outputs with human pronunciations, but there is a need for caution in interpreting matches and mismatches between model and data. We can distinguish two potential problems, which we label false positives and false negatives. False positives refer to cases where a match between model and data gives a misleading assessment of the success of the model. Such diagnostic errors can occur if the corpus contains errors, either as a result of participant error or transcription error. Indeed, it is possible to find many examples in the Mousikou et al. corpus that appear to be participant or transcription errors. Pronunciations that would probably not be considered plausible by most listeners include, for example, tamcem pronounced t{ksim, pispy pronounced pIpsi, and daxing pronounced d1ksIN. If one of the models considered by Mousikou et al. produced any of these presentations it would have been scored correct (on the basis that any output matched by a transcription in the database is correct), but we suggest that scoring a model in this way would constitute a false positive. Experiments 1 and 2 (below) will provide evidence for this claim.

False negatives refer to cases where the failure to find a match between model and data (because the model’s pronunciation is not present in the corpus) gives a misleading assessment of the failure of the model. The fundamental issue here is that it is not safe to assume that the participants’ responses exhaust all possible valid responses. Restricting the set of valid responses to those actually produced by at least one participant neglects the potential for alternative responses that may be apparent to individual readers. As noted above, Pritchard et al. criticise Perry et al.’s scoring criterion as overly lax for including pronunciation possibilities that “readers simply do not consider”. But we must also ask whether forcing participants to produce a single pronunciation of a nonword allows us to sample all of the possibilities that they do consider. A methodology that excludes plausible pronunciations may provide a biased standard to assess the performance of models. Furthermore, false negatives may also arise as a consequence of transcription choices (i.e. an erroneous transcription will result in the actual pronunciation being excluded from the corpus of ‘correct’ pronunciations), as we show now.

### Testing a different model using the corpus-based method

To illustrate some of the above issues, we applied the corpus-based method to a test of a different model of reading aloud. *Sequitur G2P* (henceforth Sequitur) is a leading grapheme-to-phoneme conversion tool algorithm based on a data-driven algorithm introduced by Bisani and Ney (2008).^2^ It is commonly used as a component of speech synthesis applications (e.g. Sawada et al.,, 2014), as well as in speech recognition applications (e.g. Panayotov et al.,, 2015), in both cases serving as a tool to generate phonemic transcriptions of words for which no dictionary entry is available to the system (e.g. proper names, toponyms, rare words, etc.). Sequitur is based on the idea of joint-sequence modelling, that is a word-pronunciation pair is modelled as a sequence of units called *graphones*, each one representing a mapping between adjacent letters to adjacent phonemes, where the maximum allowed number of symbols (letters or phonemes) on each side of a graphone is a parameter set by the user. The algorithm learns associations between sequences of graphones, the maximum length of learnable sequences also being a free parameter. These associations are trained on large phonemic dictionaries in the target language. In the current work, Sequitur was trained on a selection of 64,598 entries from the CELEX phonetic dictionary for English (Baayen et al., 1995).

Although it is not a psychological model, Sequitur may provide further insights into the strengths and weaknesses of each method that would not be detected by just testing CDP++ and RC00. Sequitur is capable of producing pronunciations for any text string, and so we were able to test it on the 915 disyllabic nonwords in Mousikou et al., (2017)’s database (see Section 1 in Supplementary Materials for further details about Sequitur and how we generated pronunciations from it). For each nonword, the model’s output was deemed correct if it matched at least one reference pronunciation. To ensure our application of Mousikou et al., (2017)’s method was correct we performed a similar assessment of the CDP++ model (Perry et al., 2010) and the RC00 rule-based disyllabic algorithm of Rastle and Coltheart (2000). Note, Mousikou et al., (2017) evaluated CDP++ and RC00 on their output pronunciations and stress assignment. Here, we focus on pronunciation only and as a consequence, our results are to be compared with those in the *Pronunciation* section in Mousikou et al., (2017).

Table 1 reports match scores for each model (i.e. the percentage of nonwords for which the model’s output matched with at least one human pronunciation in the corpus). Following Mousikou et al., (2017), we scored each pronunciation by both strict criteria (which required phoneme strings to match exactly) and lenient criteria (which allowed substitutions between short vowels and schwas). The match scores for CDP++ and RC00 replicate those calculated in Mousikou et al., (2017)^3^. Sequitur scored the worst among the three models, with a score of 70% using strict scoring and 75% using lenient scoring, though it was not far behind the CDP++ model.

**Table 1:** Matching (%) of CDP++, RC00 and Sequitur pronunciations of the 915 nonwords from (Mousikou et al., 2017) against human pronunciations from the same work

Pronunciations	1st	2nd	3rd	4th	5th	6th	7th	Match	Absent
*Strict scoring*									
CDP++	44	16	8	4	2	1	0	77	23
RC00	55	20	7	4	2	1	1	89	11
Sequitur	40	15	6	4	2	1	1	70	30
*Lenient scoring*									
CDP++	50	13	7	4	2	1	0	79	21
RC00	69	14	5	2	1	1	0	92	8
Sequitur	50	12	5	3	2	1	1	75	25

The *i*-th column reports the percentage of model/algorithm output that matches the *i*-th most frequent human pronunciation; Match is the percentage of output matching any human pronunciation; Absent is the complement of Match. Strict criteria means that two pronunciations (phoneme strings) have to match exactly, while lenient means that substitution errors between short vowels and schwa (both ways) are forgiven

#### Sequitur’s performance and the problem of false negatives

To get further insight into Sequitur’s poor performance under the corpus-based method, we looked specifically at its pronunciations that were deemed incorrect. This led us to discover a number of systematic discrepancies between the human pronunciations in Mousikou et al., (2017) and Sequitur’s pronunciation of the same nonword.

The most common discrepancy involved a 9→$ substitution:^4^^,^^5^ Many nonwords were pronounced by Sequitur with a $ phoneme (i.e. the long vowel, Ɔː, in *law, thought,* and *war*) when all human transcriptions used a 9 phoneme (i.e. the diphthong, , in *jury* and *cure*) in the same place. For example, outslaw was pronounced as 6ts1$ by Sequitur and as 6ts19 by humans). This can be explained by the fact that Mousikou et al., (2017) treated $ phonemes and 9 phonemes as equivalent and conflated them into 9.^6^ Conversely, the $ phoneme never appears in the outputs of CDP++ and RC00, and nor in Mousikou et al., (2017)’s transcriptions of human speakers. This creates an unfair assessment of Sequitur because even if its use of a $ phoneme is actually acceptable, all of Sequitur’s outputs that used $ were deemed incorrect under the corpus-based method since it was impossible for them to match with any transcriptions of human speakers. In reality, it is likely that at least some of these pronunciations by Sequitur were actually acceptable, given that similar pronunciations of real words can be found in its training set, CELEX. For example, Sequitur’s pronunciations of outslaw (6ts1$) and glorak (gl$r{k) are generalizations from the CELEX pronunciation of real words like outlaw (6tl$) and glory (gl$rI), respectively. That is, the corpus-based scoring of some of the model’s pronunciations reflects false negatives.

A number of other discrepancies between Sequitur and Mousikou et al., (2017)’s transcriptions of human speakers are described in Section 4.1 in Supplementary Materials. Note that unlike the 9→$ substitution example above, these remaining discrepancies are not due to conflation of two phonemes (none of the phonemes mentioned below were conflated in Mousikou et al.,, 2017) but they still highlight potential false negatives – cases where Sequitur may have learned acceptable pronunciations from CELEX only to be deemed incorrect under the corpus-based method. For example, many nonwords with word final ‘y’ such as pifty were pronounced with a final I by Sequitur whereas all humans pronounced pifty with a final i. Yet similar pronunciations of real words can be found in CELEX (e.g. fifty→fIftI, misty→mIstI). Similarly, Sequitur pronounced nonwords like chansem with # instead of { because the CELEX pronunciation of words like chance is J#ns and not J{ns. If Sequitur learned correctly from the training set (as the examples above suggest), then either CELEX itself is wrong or those pronunciations should be considered correct (despite not matching any transcription of human speakers in Mousikou et al.,, 2017). CDP++ may have been similarly susceptible to false negatives, since this model was also trained on CELEX. Indeed, analysis of its pattern of errors reveals errors similar to those described above (e.g. sometimes using I instead of i for nonwords with word final ‘y’). Note that RC00 uses hard-wired rules to sidestep many of these errors (e.g. its algorithm identifies word final ‘y’ as a suffix, and based on its stored rules for suffixes, applies the i pronunciation).

### A new method of testing models of reading aloud

Given the potential for diagnostic errors associated with the corpus-based method, together with the other limitations of this method, it is worth considering other methods for assessing model pronunciations. To this end, we propose a new method in which participants are asked to listen to and rate the plausibility of nonword pronunciations. This method does not require any transcription and allows researchers to directly test the plausibility of candidate pronunciations, rather than discarding these pronunciations because they do not conform to grapheme phoneme correspondence rules, or were not produced by a (relatively small) sample of human readers. The outputs of theoretical models can be produced by a human speaker, or (as is the case in the experiments described here) can provide the input to a speech synthesizer, so that the models really do name the nonwords aloud. This ratings-based method also has the benefit of being easy to implement online which makes data collection easier and possible to run entirely remotely.

In the following two experiments, we directly compare this new ratings-based method with the corpus-based method. We took the output of CDP++, RC00 and Sequitur for the nonwords from Mousikou et al., (2017), and used these outputs as the input to a speech synthesizer. The resulting sound files were played to participants one at a time along with the corresponding written nonword itself, and online participants rated how well the pronunciation matched the written nonword. We considered a model’s output appropriate if listeners judged the pronunciation reasonable given the spelling. Our key findings are: a) the corpus-based method does indeed introduce a number of mistakes, both false positives (categorising implausible pronunciations as correct) and more often, false negatives (categorising acceptable pronunciations as incorrect), b) our ratings method has a high hit rate and low false-alarm rate in assessing nonword reading accuracy, and arguably does a better job than the corpus-based method, and c) although we observe a similar outcome as Mousikou et al., (2017)’s evaluation in terms of RC00 outperforming CDP++, the two methods differ in terms of their evaluation of Sequitur, which performed much better under our ratings-based method. As we detail below, it can be argued that our method provided a more accurate description of the performance of Sequitur. Together, these findings indicate that our new method can be used to facilitate the developments of better models in the future.

## Experiment 1

The first aim of Experiment 1 was to assess the reliability of our ratings method’s assessment of nonword pronunciations. Participants were asked to rate correct (modal and minor) human pronunciations from Mousikou et al., (2017) as well as manipulated pronunciations where deliberate errors were introduced. The ratings of modal responses were used to estimate the sensitivity of the method (i.e. proportion of modal pronunciations rated as correct) and ratings of deliberate errors were used to estimate the specificity of the method (i.e. proportion of deliberate error pronunciations rated as incorrect. Ratings of modal and minor responses were used to assess whether the method could detect fine-grained differences amongst correct pronunciations, e.g. although both modal and minor pronunciations are likely to be rated as correct using binary classification, the former may yield more positive responses than the latter on the six-point scale.

The second aim was to use our ratings method to re-evaluate possible errors of the corpus-based method. For this purpose, we used the ratings method to re-assess nonword pronunciations that were deemed errors on the basis of the model’s output matching with 0 out of 41 human responses (i.e. we tested for false negatives), and to re-assess pronunciations that were deemed correct on the basis of the output matching with only one out of 41 human responses (i.e. testing for false positives). These latter items may be good candidates for false positives with the single matches reflecting a transcription error or lapses in concentration by the participant that cause implausible pronunciation.

### Method

#### Design and materials

Experiment 1 used 528 nonwords, corresponding to all of the nonwords from Mousikou et al., (2017) for which at least one of the three models (CDP++, RC00 and Sequitur) was deemed (a) incorrect (did not match with any human responses), or (b) correct, by matching with only one out of 41 human responses. We tested multiple pronunciations for each nonword. These pronunciations were distributed across six conditions. The first three conditions were designed as a test of the ratings method:

##### (i) Human Modal Pronunciation

The modal pronunciation of each nonword was determined based on its most frequent pronunciation in Mousikou et al., (2017)’s corpus. We expected that the pronunciations in this condition would receive the highest ratings.

##### (ii) Human Minor Pronunciation

A minor pronunciation of each nonword was determined by choosing a pronunciation that was produced by between two and six speakers in Mousikou et al., (2017)’s corpus (if there was more than one candidate, the more frequently produced pronunciation was chosen). It was not possible to use all 528 nonwords in this condition because some did not have a pronunciation that was shared between two and six speakers. We expected that the pronunciations in this condition would receive lower ratings than those in the Modal Pronunciation condition.

##### (iii) Deliberate Error condition

For each nonword we generated an erroneous pronunciation by changing one phoneme from the Human Modal pronunciation at random, according to the following constraints: A consonant could be substituted only by another consonant with a different place and manner, and a vowel or diphthong could be substituted only by another vowel or diphthong with a different position (front, mid, back) and length (short vowel, long vowel or diphthong). For example, i, which is a long and fronted vowel, could be substituted by U, which is short and back, but not by I, because it is also fronted; likewise, p (bilabial plosive) could be substituted by S (alveolar fricative) but not by b (also bilabial). In this way we tried to obtain errors that were unequivocal but at the same time not too distant from the written form. We expected the pronunciations in this condition to receive the lowest ratings.

The remaining three conditions – *(iv) CDP++*, *(v) RC00* and *(vi) Sequitur* – were designed as a cross-check on the accuracy of the scoring of model outputs under the corpus-based method. The pronunciations in each of these conditions were those produced by the respective models. These were pronunciations that had been produced by either no human participants (‘Zero Match’ items) or one participant (‘One Match’ items) in Mousikou et al., (2017)’s corpus. Table 2 reports the number of pronunciations per category included in our experiment.

**Table 2:** Number of pronunciations per condition in Experiment 1

Condition	Number of pronunciations
Human Modal	528
Human Minor	390
Deliberate Error	528
CDP++	279 (209,70)
RC00	151 (98,53)
Sequitur	325 (270,55)

The total for Human Minors is less than Human Modals because not all nonwords had a minor response. Totals differ for each model (Sequitur, RC00 and CDP++) because pronunciations were excluded if they matched with more than one human response. The parentheses indicate how many pronunciations matched with 0/41 human responses (first number) or 1/41 human responses (second number). For example, out of 279 CDP++ pronunciations, 209 matched with 0/41 human responses and 70 with 1/41

Using *Microsoft Speech Synthesizer*^7^, pronunciations were synthesised from either Mousikou et al., (2017)’s DISC transcriptions of human pronunciations or from the models’ DISC outputs. The British female voice “en-GB, Hazel” was used in all experiments. This synthesiser allows the user to specify the desired pronunciation of a given written stimulus in terms of phonetic transcription, as well as to control some prosodic aspects, like speech rate. Although we found this synthesiser to be better than alternative ones (e.g. *eSpeak*^8^), one limitation is the lack of control in positioning lexical stress, which, although considered by the programming interface, is ignored by the synthesizer, which places the stress according to pre-determined rules not accessible to the user. As we were not focused on stress assignment, we accepted this limitation. Note the results reported in the *Pronunciation* section of Mousikou et al., (2017) also disregarded stress assignment. The reader interested in using *Microsoft Speech Synthesizer* for their own research is referred to Section 5 in Supplementary Materials for practical suggestions.

#### Procedure

A short screening test was used before the main experiment. Here, participants completed five multiple-choice questions, each of which played aloud the correct pronunciation of an existing English word; participants were required to match the pronunciation with one of three written forms presented on the screen, e.g. hear: *crane*, choose among: *crane, frame, train*. If participants failed to get more than 3/5 correct answers, they did not progress to the main experiment.

For the main experiment, six stimuli lists were constructed, each containing 528 nonword pronunciations so that each condition of a given nonword pronunciation featured at least once across the lists (although no list contained the same orthographic form more than once and hence participants were never exposed to two pronunciations of the same nonword). An additional constraint was to ensure that each participant rated no more than 200 stimuli (to avoid boredom and fatigue), and thus, each of the six lists was randomly divided into three lists of 176 pronunciations, giving a total of 18 lists.

In addition to the material described above, we added ten catch trials to each list (the same ones for all lists). These were ten pronunciations of nonwords that were not amongst the 528 nonwords used in our experiment, but which all 41 participants from Mousikou et al., (2017) produced (and were thus highly likely to be correct). Five of them, called Accurate, were synthesised to be consistent with the human pronunciation. The other five, called Inaccurate, were distorted by the same procedure used to obtain Deliberate Errors. Participants in our experiment were expected to rate Accurate stimuli as very good and Inaccurate stimuli very bad. If this was not the case, participants were likely to be inattentive or using the wrong audio equipment.

*Gorilla Experiment Builder*^9^ was used to host the experiment online (Anwyl-Irvine et al., 2018). Each orthographic stimulus was presented while the corresponding auditory stimulus was played once. Participants could re-play the audio by pressing the space bar and rated the pronunciation by clicking on a six-point scale (*Very bad, Bad, Probably not OK, Probably OK, Good, Very good*) displayed below the orthographic stimulus. The task can be experienced online.^10^

#### Participants

Participants were recruited using *Prolific*^11^. The average completion time was 12 minutes and participants were paid 2.20 GBP (11 GBP/hour pro-rata). Criteria for selection was as follows: (i) Monolingual English-as-first-language Speakers, (ii) British citizen and resident (iii) No diagnosis of literacy difficulties (e.g. dyslexia), (iv) Prolific approval rate above 95%.

A total of 121 participants were recruited. Nine of these were rejected because they did not finish the experiment and four were rejected because they failed the initial screening task. Thus, 108 participants completed the experiment (ensuring there were six participants for each of the 18 stimuli lists). This sample size was deemed sufficient because it ensured that we obtained approximately 3000 observations per condition which satisfies recently proposed criterion for properly powered experiments of this kind (Brysbaert and Stevens, 2018; Brysbaert, 2019).

Overall, 98/108 of participants answered at least 9/10 Catch Trials correctly, and 104/108 participants answered at least 8/10 correctly, which suggests that overall participants were attentive and that the synthesiser was capable of rendering speech satisfactorily. However, upon closer inspection of the four participants that answered more than two catch trials incorrectly, we identified three of these participants as outliers: two of them rated almost all the stimuli from the main experiment as implausible, while the third one rated Deliberate Error pronunciations better than anything else and in general produced erratic responses (the fourth participant’s answers were relatively sound). Those three participants were excluded from further analyses as we believe that they either did not understand or did not attend to the task.

### Results

#### Assessing sensitivity and specificity

In this section, we assess the reliability of the ratings method by obtaining *Sensitivity* and *Specificity* estimates from participants’ ratings of Human Modals and Deliberate Errors. Sensitivity is defined as the proportion of Human Modal pronunciations rated as correct, while Specificity is the proportion of Deliberate Error pronunciations rated as incorrect.

**Fig. 1:**
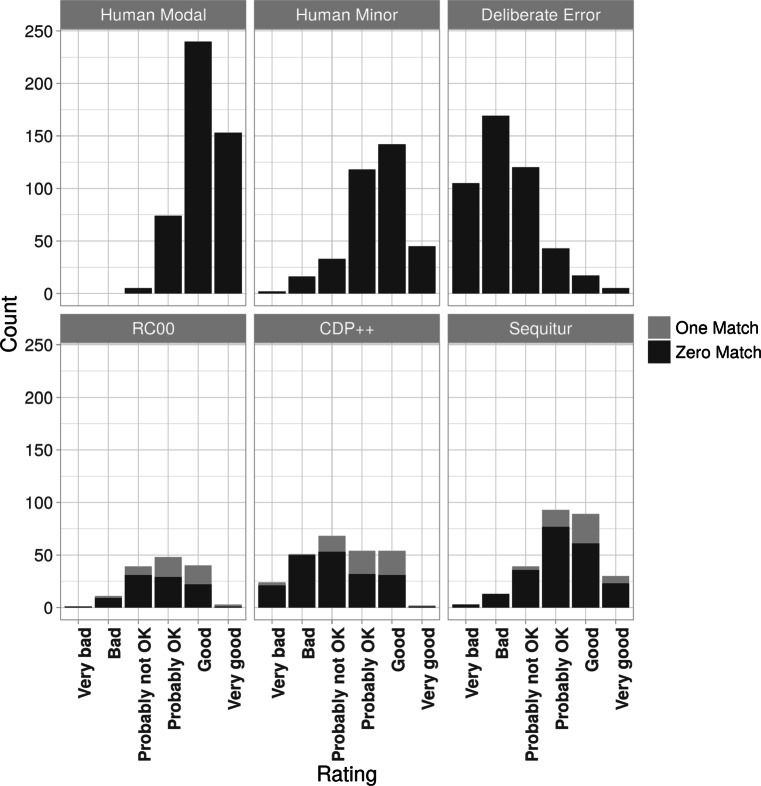
Rating counts for Experiment 1 separated by pronunciation condition and rating

We obtained one binary correct/incorrect score for each pronunciation (i.e. 528 scores for Human Modals and 528 scores for Deliberate Errors) by (i) calculating the median rating of each pronunciation (medians were used because we treated the rating scale as an ordinal rather than a continuous scale), and (ii) converting each of these median scores to a correct response (rating of “Probably OK”, “Good”, or “Very good”), or incorrect response (rating of “Probably Not OK”, “Bad”, “Very bad”). When a median score was tied between “Probably Not OK” and “Probably OK” this was rounded down to “Probably Not OK” (this was the case for eight Modal and 21 Deliberate Error pronunciations). The pattern of results is displayed in Fig. 1. Using these scores, our initial estimation of Sensitivity was 475/528 = 90*%*, and Specificity was 465/528 = 88*%*.

However, after an inspection of the 53 Human Modal pronunciations that were rated as incorrect (and which thus reduced the Sensitivity score), it became clear that sensitivity would be near-perfect were it not for the 9→$ substitution confound in Mousikou et al., (2017)’s stimuli set. As described above, the 9→$ substitution refers to the fact that in Mousikou et al., (2017)’s transcriptions of human pronunciations, $ phonemes (i.e. the long vowel, , in *law, thought,* and *war*) and 9 phonemes (i.e. the diphthong, , in *jury* and *cure*) were treated as equivalent and conflated into 9. This is relevant to our sensitivity estimate because 48 of the 53 Human Modal pronunciations that were rated as incorrect by our participants were transcribed by Mousikou et al., (2017) with a 9 phoneme when they probably should have been transcribed with a $ phoneme. Consistent with this claim, we found that for 27 of these nonwords, a Sequitur pronunciation of the same nonword used a $ phoneme in the same position that the Modal pronunciation used a 9 phoneme; crucially, all of these Sequitur pronunciations were rated as acceptable (see Table S6 in Supplementary Materials). Once we correct for this error, we obtain a sensitivity score of 475/480 = 99*%* and thus confirms our method is sufficiently sensitive for detecting plausibility of nonword pronunciations.

Furthermore, an analysis of the five remaining incorrect Human Modal pronunciations revealed that two of them, baininx and cherinx, were transcribed with a final k. A check of the original audio files^12^ revealed that speakers pronounced those two nonwords with a final ks, which is clearly more plausible. This example revealed a clear limitation of the corpus-based method’s reliance on transcriptions and further highlighted the need to cross-check transcriptions.

We conducted a similar evaluation of our initial specificity estimate (i.e. the 63 Deliberate Error pronunciations that were rated as acceptable; the full list is reported in Table S7 in Supplementary Materials). At least six of those 63 ratings can be attributed to poor design of the Deliberate Error: although these pronunciations were designed to be Deliberate Errors, their random generation unintentionally coincided with a pronunciation from another category (e.g. the Deliberate Error for the nonword outbost was generated from the Human Modal pronunciation, 6tbQst→6tb5st, but the random Q→5 substitution generated an identical pronunciation as the Human Minor pronunciation, which was also 6tb5st).

Although we could only identify six cases like this (when the error was an exact match with another pronunciation), it is likely that other Deliberate Errors were also poorly designed (i.e. although the error did not match with another pronunciation, the random generation nevertheless produced a plausible pronunciation).

Another possible source of lower specificity score is that some of these Deliberate Errors were poorly synthesized. This may have obscured the error we introduced and led participants to judge the pronunciation as acceptable. In an attempt to assess the role of the speech synthesizer in producing false-positive responses, we recruited three trained phoneticians who all had a PhD in Phonetics, were native British English speakers, and were blind to the experiment’s purpose, and asked them to verify whether each of the 63 phonemic strings had been produced accurately by our synthesizer. We used Gorilla Experiment Builder to present phoneticians with each of the 63 phonemic strings (converted from DISC to IPA) alongside an audio presentation of its synthesis. Phoneticians were asked to judge whether the phonemic string had been accurately rendered by the synthesiser using the same six-point scale employed in the main experiment and were told that positive ratings (“Probably OK” or better) would be taken to indicate that all phonemes in the string were pronounced to a satisfactory standard, whereas negative ratings (“Probably not OK” or worse) would indicate that at least one phoneme had been synthesised incorrectly. For 16 out of 63 pronunciations, the modal response from the three phoneticians was that the synthesizer had produced the phoneme string incorrectly (likely a consequence of the fact that some Deliberate Errors will naturally be difficult to pronounce). Both the error of stimulus construction and the limitations of the synthesizer we used will have artificially reduced the specificity of the corpus-based method. If we recompute the specificity while putting aside these items it is estimated at 465/512 = 91*%*. Of course the limits of the synthesizer does reduce the specificity in the current experiments, but it is not a limitation of the ratings method per se.

In sum, our on-line ratings-based experiment showed high Sensitivity and Specificity scores, it was able to discriminate between human modal and minor responses, and it revealed a systematic bias in Mousikou et al., (2017)’s transcriptions which we were not aware of. These results would be further improved if carried out in laboratory conditions and with a better speech synthesizer. This suggests that the ratings method is a reasonable measure for assessing pronunciations of disyllabic nonwords.

#### Pronunciation ratings for zero and one match items

In this section, we assess the accuracy of CDP++, RC00, and Sequitur pronunciations of the zero and one match items using our ratings method. As noted above, these are the words that are most likely to have been misclassified by the corpus-based method, with zero match pronunciations being candidates for false negatives (if a pronunciation does not match exactly with any pronunciations from the reference list of 41 participants, this is not necessarily an erroneous pronunciation) and one match pronunciations candidates for false positives (a match with only one of 41 participants may reflect the fact that this participant mispronounced or that it was mistranscribed). Based on the 9→$ confound described earlier, we decided to exclude all orthographic stimuli for which any pronunciation contained the 9 and/ or $ phoneme (95/528 nonwords were excluded).

In Fig. 1, top row, we report ratings for the Human Pronunciations and Deliberate Errors, corresponding to the Sensitivity and Specificity scores above. The bottom row of Fig. 1 reports the ratings of each model: darker shades of grey denote ‘Zero Match’ pronunciations whereas lighter shades of grey denote ‘One Match’ pronunciations. Strikingly, over half of the Zero Match pronunciations (58%) were rated as correct (“Probably OK” or better) despite the fact that all of these pronunciations were rated as incorrect under Mousikou et al., (2017)’s criterion (i.e. evidence for false negatives in the corpus-based method). Notable examples include freacely (frislI), conglist (k@nglIst), and afflave (@fl1v), which all used substitutions identified earlier (e.g. i→I, Q→@, {→@) thus supporting the notion that these generalizations learned from CELEX were indeed correct. Turning our attention to ‘One Match’ pronunciations (shaded in lighter grey), these were judged more consistently with the corpus-based method (most were judged positively) but there were nevertheless some inconsistencies between the methods (19% were judged negatively). Notable examples include surbeft (s3bft), conclise (kQn2sz), pilprem (pIspr@m), and udgement (v_im), which were all judged as “Bad” or “Very bad” by our participants. Since all of these are clearly bad pronunciations, they highlight examples of when Mousikou et al., (2017)’s reference list included either transcription errors or unreliable human responses that made their method susceptible to false positives.

In sum, the ratings method of the zero match and one match items suggests that the corpus-based method is prone to making false-negative errors and false-positive errors. The false-negative errors may simply reflect the fact that the responses of the 41 participants did not produce all plausible pronunciations of these nonwords, and the false-positive errors reflect the fact that matching one response out of 41 participants is not sufficient grounds for characterizing a pronunciation as correct (people make mistakes, and matching a mistake does not entail a correct pronunciation). Combined with the high sensitivity and specificity results of the ratings method, and the fact that the ratings method picked up a transcription error that the corpus-based method was blind to, the ratings method seems a promising method for evaluating model productions that can easily be applied to any item. In Experiment 2, we compare the success of CDP++, RC00, and Sequitur models in naming the same set of disyllabic nonwords using the corpus-based and ratings-based methods.

## Experiment 2

In Experiment 2, a new group of participants was asked to rate the output of the three nonword naming models on the same set of 803 nonwords from Mousikou et al., (2017) (not just the 528 from Experiment 1 that matched with ≤ 1 human responses)^13^. They were also asked to rate the responses of three different speakers from Mousikou et al., (2017) who named the nonwords in different manners. The ratings of the model outputs and the ratings for the responses for the three speakers were compared.

### Method

#### Materials

Experiment 2 used seven pronunciation conditions: (i) CDP++, (ii) RC00, (iii) Sequitur, (iv) Deliberate Error, (v) Modal Speaker, (vi) Typical Speaker, and (vii) Outlier Speaker. Conditions (i) to (iv) are the same as their namesakes in Experiment 1 except that we include each model’s pronunciation of 803 nonwords. In contrast to Experiment 1, we also used pronunciations by individual human speakers (Conditions v-vii), as opposed to artificial categories like Human Modal and Human Minor, which are not necessarily representative of any individual speaker. To select these conditions, we employed the *Surprise Index* (SI) (Good, 1956), a statistic based on information theory that quantifies how unexpected an outcome is based on the available outcome probabilities (Siegelman et al., 2020). This measure was applied on each pronunciation of each speaker, providing 41 speaker profiles each one describing how the production of a particular speaker departs from the majority, i.e. how ‘surprising’ it is. The three selected profiles were the least surprising (Modal), the most surprising (Outlier) and the median surprising (Typical). It turns out that the Modal Speaker corresponds to the speaker who produced the modal pronunciation most often, while the Outlier is the one who produced unique pronunciations most often, thus justifying the use of SI. A formal definition of SI and a detailed description of how SI was computed on the pronunciations in Mousikou et al., (2017) is provided in Section 7 in Supplementary Materials. As in Experiment 1, all stimuli were synthesized using Microsoft Speech Synthesizer from either (Mousikou et al., 2017)’s DISC transcriptions or the models’ DISC outputs.

#### Design & procedure

Experiment 2 used 803 nonwords, and a total of 3086 unique pronunciations (sometimes, a nonword had identical pronunciations in multiple categories, so the total was not 803 multiplied by the number of categories). To ensure lists were an appropriate length for participants, stimuli were divided into 16 lists of 192 or 193 pronunciations (no nonword was used more than once within a list). The same screening task was used as Experiment 1, as were the same conditions to present stimuli. A full demonstration of Experiment 2, as well as all materials, is available online.^14^

#### Participants

A total of 155 participants were recruited, seven of which were rejected for failing the screening task and 20 of which were rejected for not finishing the experiment. Thus, 128 participants completed the experiment, ensuring there were at least eight participants for each item of the 16 lists. Recruitment strategy, sample size justification, and exclusion criteria were the same as in Experiment 1. The average completion time was 14 min and participants were paid 2.34 GBP (10 GBP/h pro-rata).

### Results

#### Comparing models using the ratings method

**Fig. 2:**
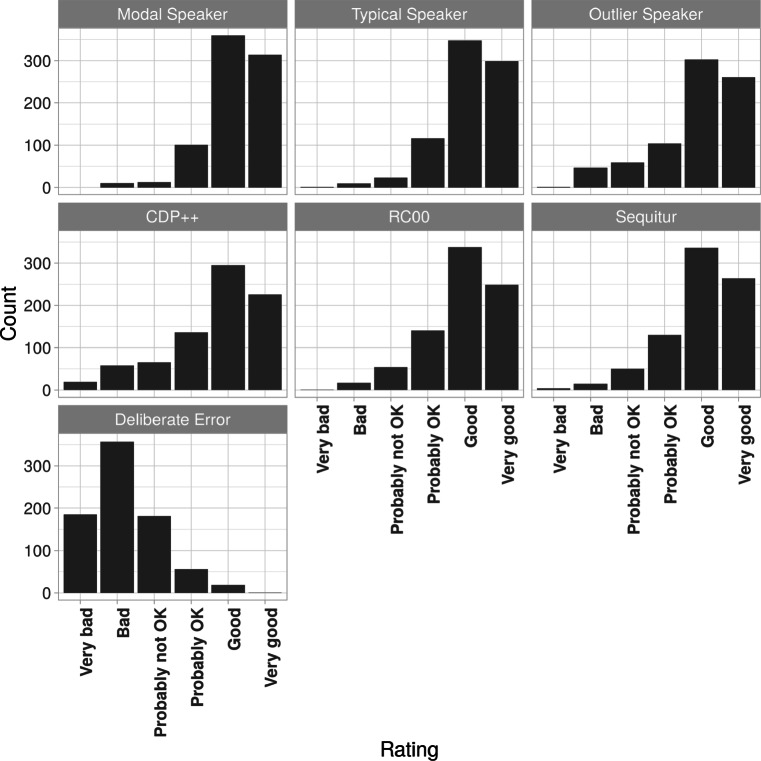
Rating counts for Experiment 2 separated by pronunciation condition and rating

Figure 2 reports the median ratings for nonwords, separated by Condition (CDP++, RC00, Sequitur, Modal Speaker, Typical Speaker, Outlier Speaker, Deliberate Error). Four nonwords (out of 803) were excluded from the analysis since the median ratings of the three human speakers’ pronunciations (Modal, Typical, Outlier) were all below “Probably OK” and hence we suspected some specific problems related to their transcription or synthesis. These were baininx and cherinx, whose transcriptions were identified as wrong, combire, which was poorly rendered by the synthesiser due to a final @r, and udstame, for which we cannot find an explanation.

Two mixed-effects regression models were conducted to test for statistical differences between each condition. Both models serve the same purpose, but one is a logistic regression model^15^ which predicts binary ratings (correct/ incorrect) and the other one is a cumulative link model^16^ that predicts ordinal-scale ratings. Both models used *Condition* as fixed factor and treated *Participant* and *Nonword* (i.e. the orthographic stimulus) as random intercepts. A by-participant random slope for *Condition* was fitted for the logistic model but did not converge for the ordinal model. BIC tests proved that all the terms in both models are justified.

**Fig. 3:**
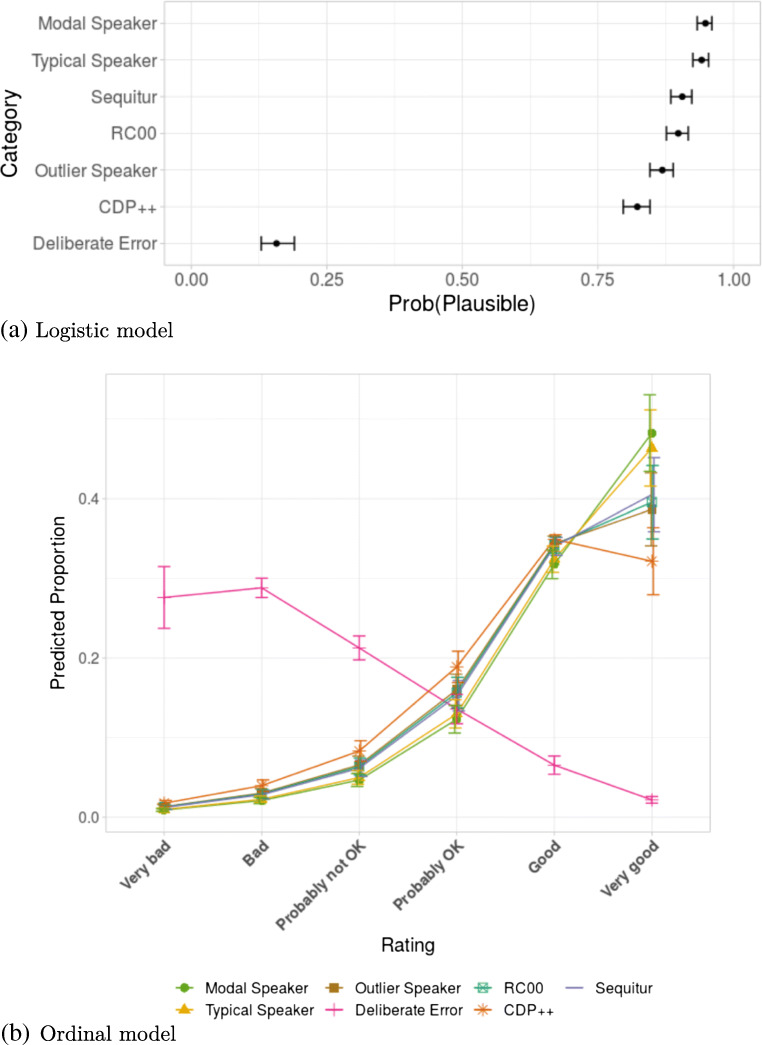
Predictions (estimated marginal means) and confidence intervals from the linear mixed-effect models fitted on the data from Experiment 2

Fig. 3a illustrates the logistic regression model by displaying the predicted probability (estimated marginal means^17^) that the model has estimated for each condition (i.e. the probability of each condition getting a plausible rating). As can be seen, the Modal Speaker had a very high probability score of 0.95, immediately followed by the Typical Speaker with 0.94, then RC00 and Sequitur both at 0.90, the Outlier Speaker 0.87 and CDP++ at 0.82. Finally, the Deliberate Error condition had a much lower probability of 0.15. To test whether these differences were significant, we used Tukey-adjusted pairwise comparisons, which compared each condition against the other. All conditions were significantly different from each other (all *t* test *p* values < 0.0001) except between Modal Speaker and Typical Speaker, and between RC00 and Sequitur. Perhaps most interestingly, RC00 and Sequitur both performed significantly better than the Outlier Speaker, which itself outperformed CDP++.

The results from the ordinal model (Fig. 3b) provides a more detailed picture by calculating the predicted proportions of each ordinal rating (“Very good”, “Good”, “Probably OK”, “Probably not OK”, “Bad”, “Very bad”) for each condition. Notably, the predicted proportions of “Very good” ratings for Modal and Typical Speaker at around 0.47, followed by Sequitur, RC00 and the Outlier Speaker at around 0.39, then CDP++ with 0.32. This confirms that two of the three models (RC00 and Sequitur) produced pronunciations that were judged within the same range of acceptability as pronunciations by real human speakers.

#### Comparing methods

Table 3 compares the ratings and the corpus-based evaluations. As can be seen in the table, the two methods provided similar assessments of the RC00 and CDP++ models, whereas performance of Sequitur was judged to be lower on the corpus-based (76% on strict criterion) compared to the ratings (90%) method. The discrepancy with Sequitur provides us an opportunity to test which method is doing a better job in evaluating model performance.

**Table 3:** Comparison of model evaluations under corpus-based methods (i.e. % match score) and ratings-based methods (i.e. % judged as acceptable)

	Corpus-based	ratings
	(strict; lenient)	
RC00	90%; 93%	90%
CDP++	78%; 80%	82%
Sequitur	76%; 81%	90%

To allow the fairest comparison, match scores from the corpus-based method in Table 1 were recomputed on the basis of the 799 nonwords analysed in 3: Sequitur’s performance improved as a consequence of eliminating the false negatives introduced by the 9→$ substitution

In a first attempt to understand the different outcome we collected an independent assessment of the acceptability of the discrepant naming outcomes. The same three phoneticians from Experiment 1 saw the orthographic string of all the discrepant nonwords alongside their corresponding phonemic strings (converted from DISC to IPA) and were asked to judge whether this was an acceptable pronunciation (note that judging the acceptability of phonemic strings rather than audio files removes any potential influence from the synthesiser on acceptability). The phoneticians’ median judgement was significantly more likely to agree with the outcome of the ratings method than the corpus-based method (57 vs. 43%), *χ*^2^= 6.78, *df* = 1, *p* = 0.004, and this effect did not differ between models (*χ*^2^= 0.90, *df* = 2, *p* = 0.63). If we adopt the phoneticians’ median judgement as ground truth for the discrepant items and combine with items that the methods agreed upon, we obtain the following values for model pronunciation acceptability: 79% for CDP++, 93% for RC00 and 87% for Sequitur. This pattern of results is more similar to the estimate of the ratings method and suggests that the ratings method provided a more accurate assessment of the performance of Sequitur.

**Fig. 4:**
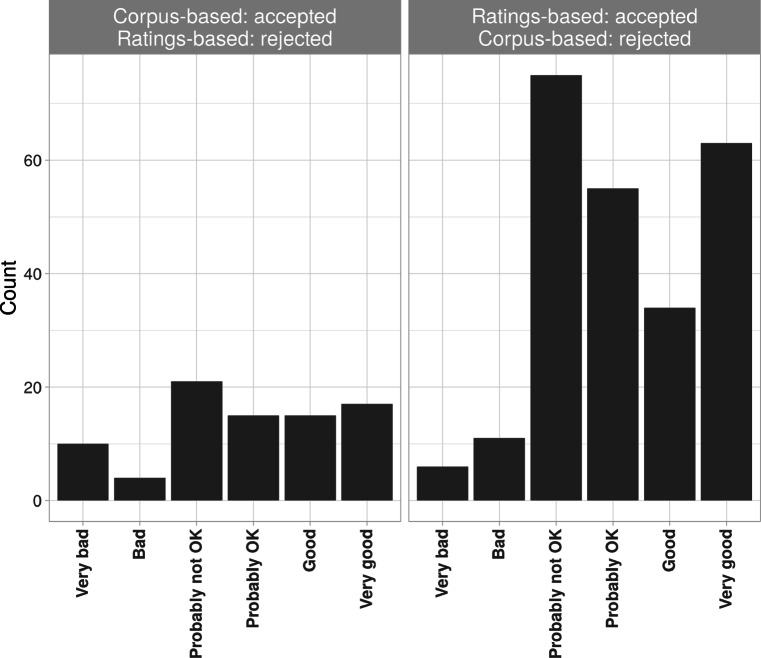
Phoneticians’ median rating counts for *discrepant* items of Experiment 2, separated by rating and by type of discrepancy, i.e. accepted under the corpus-based method (hence rejected under ratings, *left*) or accepted under ratings (hence rejected under the corpus-based method, *right*)

Figure 4 provides a closer look at phoneticians’ median ratings; the left panel shows the distribution of phoneticians’ median ratings for discrepant items that were accepted under the corpus-based method (and rejected under the ratings method) where as the right panel shows phoneticians’ median ratings for discrepant items accepted under the ratings method. The higher overall count of items in the right panel reflects the fact that there were more discrepant items rated acceptable under the ratings method. Importantly, 62% of the items accepted by the ratings method were also accepted by the phoneticians, with very few rated “Bad” or “Very bad”. By contrast, for the items accepted by the corpus-based method, there was no clear pattern with the phoneticians’ judgements. Again, this suggests that the phoneticians agreed more with the ratings compared to the corpus-based method.

It is also interesting to note that phoneticians’ judgements on the discrepant items were closely related to the typicality of the pronunciations, as measured through the surprise index (SI). The discrepant items accepted under the corpus-based method but rejected by the phoneticians had a high mean SI of 3.0, whereas those items accepted by the phoneticians had a low mean SI of 1.9 (one-sided *t* test yielded *t* = -4.3, *df* = 71.2, *p* value < 0.0001)^18^.

This suggests that discrepant items with a high SI reflected false positives from the corpus-based method: When participants from Mousikou et al. produced a rare pronunciation (a word with a high SI), it was rejected by both the ratings method and the phoneticians because it was likely a mistaken production or transcription error.

Although the above analyses suggest the ratings method was more accurate overall, it is still not clear why Sequitur fared so much better under the ratings method than the corpus-based method (see Table 3).^19^ One possibility is that some of the discrepant nonword productions were poorly synthesized, with more subtle errors missed in the ratings method. If this occurred more often for the nonwords produced by Sequitur it might help explain the results. Note, the previous analyses do not rule this possibility out as the phoneticians rated phonemic text string rather than the synthesized outputs.

To explore this possibility we asked the three phoneticians to verify the synthesiser’s pronunciations. As in Experiment 1, phoneticians were asked to judge whether the phonemic string of each discrepant item (converted from DISC to IPA) had been accurately rendered by the synthesiser on the same scale used in the main experiment, with positive ratings (“Probably OK” or better) indicating that all phonemes in the string were pronounced to a satisfactory standard and negative ratings (“Probably not OK” or worse) indicating that at least one phoneme had been synthesised poorly. Only 3% of discrepant items were unanimously rated as poorly synthesised (the remaining 97% were rated positively by at least one phonetician). However, when using the median of the three phoneticians’ ratings as the outcome (as opposed to the unanimous rating), 15% of the discrepant items were rated as poorly synthesised.^20^

Importantly though, no model was affected differently by poor synthesis when considering all discrepant items (*χ*^2^ = 1.45, *df* = 2, *p* = 0.49) or when specifically considering discrepant items rejected by the corpus-based method (*χ*^2^ = 0.60, df = 2, *p* = 0.74).

Finally, we performed a detailed analysis of the discrepant items using the same methods employed above in order to gain some additional insight. We identified 19 phonemic patterns that appeared in at least five of the discrepant items, and these are listed in Table S8 in Supplementary Material, where each pattern is analysed separately. Here we summarise the main findings.

Other, albeit less numerous cases are highlighted in Section S8 of Supplementary Material, which demonstrate the unfair penalisation of some typical Sequitur pronunciation patterns. However, some sporadic counter-evidence can be found as well. For example, g→_ in geveld, I→2 in etind and oppind and {→1 in apreds, are examples where Sequitur generalised from CELEX (see Section S8 in Supplementary Material for details), but these pronunciations were accepted under ratings and rejected by phoneticians, thus in agreement with corpus-based methods. In cases like these it remains unclear why some generalisations from CELEX are rated negatively by phoneticians but further investigation on this issue is beyond the scope of this work.

To summarise, the phoneticians’ ratings of nonword pronunciations suggest that the ratings method was more accurate in assessing the Sequitur’s performance, and the pattern of errors analysis identified some frequent pronunciation patterns by Sequitur that originate from CELEX that were deemed acceptable by the ratings method phoneticians but not by the corpus-based method. These seem to an example of false-negative errors associated with the corpus-based method with acceptable responses rejected on the basis that they did not match a reference set of pronunciations (generated from a limited set of 41 participants).

## General Discussion

In this paper we have compared two different methods of assessing disyllabic nonword naming performance. For the corpus-based method we relied on the nonword dataset of Mousikou et al., (2017) that includes 915 disyllabic nonwords and the pronunciation transcriptions of 41 human speakers. According to this method, a model is correct if its output matches at least one human transcription. Apart from providing a measure of model performance, a key contribution of the corpus-based method is that it can document the extreme variability of human nonword naming responses (e.g. in Mousikou et al., (2017)’s paper, the mean number of different pronunciations per nonword was 5.9, ranging from 1 to 22) and thus, the method is particularly important for determining factors that predict consistency or variability of pronunciations. Still, there are some limitations to the corpus-based method, including that it is extremely resource intensive, making it a challenge to expand the dataset to many more nonwords as required for future model development.

We compared this corpus-based method to a ratings-based method that makes it much easier to assess the outputs of a model by asking human participants to judge its nonword pronunciations when produced by a speech synthesizer. Despite the relative simplicity of this method, it was at least as good in characterizing the nonword naming performance of two psychological models RC00, CDP++ considered by Mousikou et al., (2017) as well as a freely available commercial text-to-speech product called Sequitur. Indeed, the ratings method: (i) identified limitations of the corpus-based method that led to errors in classifying nonword pronunciations (both false positive and false negatives), (ii) correctly assessed the accuracy of nonword pronunciations with high sensitivity and specificity, and (iii) reached a similar conclusion regarding the successes of the RC00 (Rastle and Coltheart, 2000) and CDP++ (Perry et al., 2010) accounts of word naming, (iv) did a better job in assessing the performance of the Sequitur model (see Table 3). Implications of these findings are discussed below.

With regards to the limits of the corpus-based method, we have identified three sources of error that result from the assumption that a pronunciation is only correct if it matches a transcription from a reference list of human pronunciations. First, there were transcription errors. Mousikou et al., (2017)’s reference list was transcribed by just one person (who transcribed over 37K responses) and the inevitable mistakes were treated as a correct pronunciation under the corpus-based method. Second, there were (inevitable) human pronunciation errors that were treated as correct pronunciations under the corpus-based method. Examples of transcription errors and human pronunciation errors were identified above (e.g. surbeft →s3bft, conclise→kQn2sz, pilprem→pIspr@m, and udgement→v_im).^21^ Third, the reference list only contains a limited sample of human responses (41 speakers in this case), and in some cases, there were potentially plausible pronunciations of a nonword that were not produced by participants (or which were excluded from the list because erroneous transcriptions prevented the actual pronunciations from appearing). For example, Experiment 1 highlighted numerous examples of model outputs that did not match with any human pronunciation (‘Zero Match’ pronunciations), but nevertheless were given positive ratings in the ratings method. Although we acknowledge some of these cases may be considered borderline acceptable, many outputs obtained a median rating of ’Very good’ and are thus clear examples of false negatives in the corpus-based method (e.g. freacely→frislI, conglist→k@nglIst, and afflave→@fl1v).

We also made a closer inspection of the discrepant items in an attempt to further gauge the reliability of each method. First, we demonstrated that for the majority of these items, the synthesiser had rendered the pronunciation accurately, thus lending credibility to participants’ judgements of these pronunciations. Second, we asked phoneticians to judge the phonemic string of the model pronunciation (independent from a synthesiser) and found that they were more likely to agree with the outcome of the ratings method than the corpus-based method. The phoneticians also confirmed many cases of false negatives under the corpus-based method, such as the examples outlined above (frislI, k@nglIst, @fl1v), which were unanimously deemed acceptable by the phoneticians. We also examined whether there were any common patterns of error that could provide insight into the discrepancies between methods. Notably, some of Sequitur’s outputs that were deemed incorrect under the corpus-based method (but correct under the ratings method and by phoneticians) were based on generalizations from the CELEX training set, such as i→I, {→#, and kQn→k@n. That is, the model had generalized correctly from their training set, listeners (and trained phoneticians) considered these productions appropriate, but the fact that the productions were not consistent with the limited sample of human pronunciations in Mousikou et al., (2017) meant that they were deemed incorrect under the corpus-based method.

It is difficult to remedy the three sources of error associated with the corpus-based method, outlined above. Participant errors are difficult to avoid but transcription errors may be reduced by using more than one transcriber and cross-checking these transcriptions, although this would be time consuming and expensive. To reduce its susceptibility to the third source of error (i.e. the limited sample of human responses), the corpus-based method would require a huge amount of human participants in order to capture as many pronunciation variants as possible. However, increasing the number of human participants would lead to an inevitable increase in transcription errors and human pronunciation errors.

The ratings method sidesteps the three sources of errors outlined above because there is no reliance on constructing reference lists of human pronunciations and thus, there is no inherent risk of wrongly penalising pronunciations because they do not match a given set of references (false negatives), or wrongly accepting pronunciations because they match a specific reference that is actually erroneous (false positives). Furthermore, the ratings method is much easier to implement because (i) there is no need to create a reference list of human pronunciations each time a new dataset of nonwords are introduced, and (ii) data can be collected online which is much faster and relatively cheap. Nevertheless, we acknowledge that the ratings method has its own limitations and it is important to consider how these may be addressed for future implementations. For example, although there was occasionally inaccurate synthesis in the current implementation, this can be fixed as technology improves. Furthermore, we have made our full dataset available for further analyses to facilitate further analyses of the discrepant items and thus provide further insights into the strengths and weaknesses of each method (for example, original sound files can be examined to assess which discrepant items really were transcription errors and which really were never pronounced by the human participants). The full data set may also be used for development of future computational models as a benchmark for capturing the fine-grained and heterogeneous range of acceptable pronunciations for each pseudoword (e.g. Schmalz et al.,, 2020; Ulicheva et al.,, 2021).

In summary, generalisation to unfamiliar nonwords is a key challenge for models of word naming. Datasets such as that collected by Mousikou et al., (2017) are valuable for testing models, and particularly for helping to explain the variability of human pronunciations. However, comparing model output with human pronunciations is not straightforward. The production and corpus-based method for assessing model performance is extremely labour intensive, making it difficult to test models on a wide range of nonwords beyond existing datasets. In addition, we have identified a number of limitations with the accuracy of this method. By contrast, the ratings-based approach is an easy-to-implement alternative which is not prone to the same mistakes as the corpus-based method, and is arguably a more accurate test of model performance. We hope this method will be useful in future model development.

## Electronic supplementary material

Below is the link to the electronic supplementary material.

(PDF 1.30 MB)

## Footnotes

1 This is true even for published papers where authors included stricter scoring criteria, such as Seidenberg et al., (1994), who report an experiment in which they supplemented their analogy-based method with a criterion that tested whether the model’s pronunciation matched one of the top three pronunciations given by a sample of 24 participants. Seidenberg et al., (1994)’s appendices report the most frequent pronunciation for each of their nonwords, but not what the second and third most common pronunciations were, and thus it is not possible to score other models against the same data set.

2 Sequitur is freely available from https://www-i6.informatik.rwth-aachen.de/web/Software/g2p.html

3 There are minor discrepancies between our match scores and those reported in Mousikou et al., (2017), possibly due to different rounding procedures.

4 To be consistent with Mousikou et al., (2017), the present paper uses the DISC phonemic transcription system from CELEX (Baayen et al., 1995) (which was used by all three models) as opposed to the more commonly known IPA. Section 3 in Supplementary Materials provides a full transcription table for IPA and DISC phonemes.

5 In this work, single and multi-phonemic edit patterns $X\rightarrow Y$ were discovered automatically by adapting algorithms from string matching and information retrieval. These are illustrated in Section 2 in Supplementary Materials.

6 After discovering this issue, we contacted the authors of Mousikou et al. who confirmed our explanation.

7 https://docs.microsoft.com/en-gb/previous-versions/office/developer/speech-technologies/hh361644(v=office.14)

8 http://espeak.sourceforge.net/index.html

9 www.gorilla.sc

10 https://gorilla.sc/openmaterials/102572

11 www.prolific.ac

12 the Authors of Mousikou et al. kindly granted us access to the original recordings.

13 ideally, all 915 nonwords from Mousikou et al., (2017) would be used but for the reasons outlined already, we excluded the 112 nonwords affected by the 9→$ substitution, leaving 803 nonwords.

14 https://gorilla.sc/openmaterials/101478

15 computed with the glmer function from the *lme4* package in *R* (Bates et al., 2015)

16 computed with the clmm function from the *ordinal* package in *R* (Christensen, 2019)

17 computed with the *emmeans* package in *R* (Lenth, 2019)

18 The SI difference is remarkable, considering that the highest possible SI value for a distribution of 41 speakers is 5.19 (see Section 5 in Supplementary Materials)

19 One reviewer suggested the reason why Sequitur fared better under the ratings method may be that it benefited from a lax criterion (i.e. the binary correct/incorrect definition includes ‘Probably Ok’ ratings as correct pronunciations). To address this claim, we conducted additional analyses after applying a stricter criterion to the ratings method (where the definition of correct/incorrect includes only ‘Good’ and ‘Very Good’ ratings as correct pronunciations - and when a subset of the most lenient participants are excluded); under this criterion, rankings of the seven pronunciation conditions under the ratings method did not change (Sequitur was still ranked third highest, above RC00, CDP++, the Outlier Speaker and Deliberate Errors), thus demonstrating that Sequitur’s improved performance under the ratings method was not due to lax ratings. Further details of these extra analyses are available by request.

20 Although the figure of 15% may seem quite high, it is important to note that only 2% were rated as ”Bad” or ”Very Bad”, and these poor ratings only refer to the discrepant items (this is not a measure of the overall quality of the synthesizer). But of course, a better synthesizer can only improve the ratings method.

21 Although these examples are clearly incorrect and do not belong on the reference list (all were rated as “Very bad” by our participants), it is difficult to know whether these are transcription errors or human pronunciation errors.

## Declarations

### Open Practices Statement

The experiments were not preregistered. All of the materials from the experiments are available as specified in the relevant sections of the manuscript. R code is available from the first author by request.
