# Supplementary material for: Does that sound right? A novel method of evaluating models of reading aloud: Rating nonword pronunciations
Source: Behav Res Methods. 2022 Jun 1;55(3):1314–31. doi: 10.3758/s13428-022-01794-8 (PMC10126079; doi:10.3758/s13428-022-01794-8)
Supplement: Supplementary file 1 — (PDF 1.30 MB) [file 13428_2022_1794_MOESM1_ESM.pdf]

Supplementary material: Does that sound right? A novel method of evaluating models of reading aloud

Michele Gubian
Institute of Phonetics and Speech Processing, LMU Munich, Germany
Ryan Blything
School of Psychology, Aston University, UK
Colin J. Davis and Jeffrey S. Bowers
School of Psychological Science, University of Bristol, UK

1 Sequitur

1.1 The algorithm

Sequitur is based on the idea of joint-sequence modeling, that is a word-pronunciation pair is modeled as a sequence of units called *graphones*, each one representing a mapping between adjacent letters to adjacent phonemes. For example, the mapping between the word 'mixing' and its pronunciation `mIksIN` can be modeled as a sequence of four graphones as follows:

(m, `m`) (i, `I`) (x, `ks`) (ing, `IN`),

where each graphone is a many-to-many association of letters to phonemes. Such a grouping is called a *co-segmentation*. Clearly there are many other ways of co-segmenting a given word-pronunciation pair. For example,

(m, `m`) (i, `I`) (x, `k`) (-, `s`) (i, `I`) (n, -) (g, `N`),

where '-' denotes the empty symbol, is also a valid co-segmentation for (mixing, `mIksIN`).

Joint sequences make it possible to define the problem of determining the pronunciation of a given word as a maximisation of probability over sequences of graphones, which has several advantages in terms of algorithm formulation. Formally, if w is a word, we want to choose the pronunciation ϕ^* that maximises the conditional

M. Gubian and R. Blything are co-first authors.

probability $p(\phi|w)$, which is equivalent to maximising the joint probability $p(w, \phi)$, since w is fixed:

$$\phi^* = \underset{\phi}{\operatorname{argmax}}\, p(\phi|w) = \underset{\phi}{\operatorname{argmax}}\, \frac{p(w, \phi)}{p(w)} = \underset{\phi}{\operatorname{argmax}}\, p(w, \phi).$$

Let $S(w, \phi)$ define the set of all possible co-segmentations of a given $(w,\ \phi)$ pair. Then $p(w, \phi)$ can be expressed as the sum of probabilities of all possible graphone sequences associated to it:

$$p(w, \phi) = \sum_{q \in S(w, \phi)} p(q),$$

where $q = (q_1, q_2, \ldots, q_K)$ is a graphone sequence. The probability of q is modeled using a standard M-gram approximation, i.e. the probability of q_i is assumed to depend only on the preceding $M - 1$ graphones:

$$p(q) = \prod_{i=1}^{K} p(q_i|q_{i-1}, \ldots, q_1) \cong \prod_{i=1}^{K} p(q_i|q_{i-1}, \ldots, q_{i-M+1}).$$

In this way, the estimation of all $p(q)$ requires only the availability of enough training examples of graphone sequences of length M, where M is chosen by the user. Note that the length of a graphone sequence alone does not say anything about the length of the corresponding letter or phoneme strings, as each graphone q_i can in principle span an arbitrary length on either side. In practice, minimum and maximum number of symbols are fixed at training time, usually between 0 and $L \geq 1$ on both sides (excluding the $(0, 0)$ combination). The estimation of $p(q)$ is carried out by maximum likelihood using the well-known Expectation-Maximisation algorithm (EM, Dempster, Laird, & Rubin, 1977) applied to a training set of word-pronunciation pairs (i.e. a dictionary) where no prior segmentation is required. In order to prevent over-fitting, procedures known as smoothing and trimming are also applied on top of the pure maximum likelihood estimation (see Bisani and Ney (2008) for details).

1.2 Training

In this work, Sequitur was trained on the CELEX phonetic dictionary for English (Baayen, Piepenbrock, & Gulikers, 1995). The training material was selected as follows. First, all dictionary entries containing any character other than lower-case letters were excluded, as this was the safest way to remove most of the acronyms, abbreviations and errors. After removing duplicates, the usable dictionary contained 64,598 entries. The lexical stress symbol was removed from the phonemic transcriptions, as this work is focused on pure grapheme-to-phoneme conversion. Finally, the dictionary was split randomly into a training set and a test set at 90%-10% proportion.

In order to train Sequitur, one has to specify a number of settings. In this work, all settings were kept at their default value except for the two main parameters M and L, which identify the length of the M-gram approximation and the maximum number

of symbols allowed on each side of a graphone (minimum being zero), respectively (see section 1.1). We explored both parameters on a grid where M was varied from 1 to 5 and L from 1 to 4. Of the 20 combinations, $(M = 5, L = 4)$ was excluded as training did not complete after over two days. Table S1 reports pronunciation error rates estimated on the test set (around 6,500 words) for all the parameter combinations. We can spot a number of clear trends. First, the 'naïve' setting $(M = 1, L = 1)$, which

	$M = 1$	$M = 2$	$M = 3$	$M = 4$	$M = 5$
$L = 1$	98.25	66.15	30.60	13.75	**9.58**
$L = 2$	85.93	23.58	12.07	10.99	10.94
$L = 3$	67.29	17.89	16.30	16.24	16.24
$L = 4$	50.71	22.89	22.63	22.63	–

Table S1
Pronunciation error rates (%) estimated as ratio of mismatching pronunciations on a test set of around 6,500 *words from the CELEX dictionary that was not used during training. L is the maximum length of a graphone on either side (minimum is zero), M is the M-gram length. In bold the best solution.*

maps single letters to single phonemes without taking context into account, performs really badly. Performance rapidly improves as more and more context $(M > 1)$ is taken into account, reaching the top at $(M = 5, L = 1)$. Allowing larger graphones $(L > 1)$ is beneficial for shorter context, but for a given L a plateau is reached as M increases where error rate is higher for higher L. Moreover, training time increases enormously with L (and probably training never converges for too high M and L). We interpret the trend on L as caused by the limited size of the training set, as well as the limited length of words and pronunciations. For example, the $(M = 5, L = 4)$ model ideally would require several examples of mapping of up to five consecutive groups of four letters or phonemes, i.e. words or pronunciations of length up to 20 symbols, which are very rare in the English language.

Based on the results in Table S1, we selected the $(M = 5, L = 1)$ model. We did not re-train the algorithm on the whole 100% of the usable dictionary as otherwise we would not be able to provide an estimate of accuracy to compare with the evaluations on nonwords.

2 Automatic discovering of single- and multi-phonemic patterns of error

This section describes two procedures we developed in order to automatically extract patterns of error at the phonemic level from pairs of pronunciations, each pair being composed of a target phonemic string, e.g. produced by a model, and its associated reference, e.g. the modal human pronunciation. By pattern of error we mean any single edit operation, i.e. substitution, insertion or deletion, or a sequence thereof. For example, given the target pronunciation `b#s@l` and its reference version

`b{st@l` (for the nonword BASTLE), the task is to automatically extract: `{`→`#` (substitution) and `t`→ (deletion). Being able to extract such patterns automatically and as accurately as possible is an invaluable asset when large amount of pronunciations have to be analysed, like in this work.

The first procedure is an adaptation of the well-known Levenstein algorithm for string alignment (Levenshtein, 1966) where prior knowledge in the form of a hierarchy of phonemic classes guides the purely symbolic Levenstein algorithm to produce more plausible results. The second procedure uses the SPADE algorithm (Sequential PAttern Discovery using Equivalence classes, Zaki, 2001) in order to identify adjacent edit errors that tend to co-occur. This allows to find patterns that should be considered as a whole, e.g. when a nonword like BESHOM is produced as `bEshQm` instead of `bESQm`, the substitution `S`→`s` is not independent of the following insertion →`h`, but rather they both originate from processing the SH letter sequence.

Importantly, neither of the two procedures makes use of the orthographic form the pronunciations are realisations of. This means that the first procedure should be able to identify e.g. the edit operation `t`→ without knowing that the orthographic stimulus contained a T or the context STL; similarly, the second procedure should identify e.g. the frequent edit sequence `S`→`s` followed by →`h` without knowing that the orthographic stimulus contained SH. The reasons for not using the orthographic information are mainly simplicity of implementation, generality and scalability. In fact, using orthography to somehow infer what type of error a pronunciation model made ultimately requires one to use information about how the model works, e.g. if it is rule-based it requires one to identify which set of rules triggered a given pronunciation from an orthographic input. This is clearly a model-specific approach, whose complexity depends on the model's complexity, and is neither general nor scalable. We opted for a less powerful but simpler and more scalable approach.

The R code implementing the procedures described below is available on request from the first author.

2.1 Phonemic class-aware alignment

Suppose we have a target pronunciation t = `b#s@l` and its reference version r = `b{st@l`, both realisation of the nonword BASTLE. If we apply the Levenstein algorithm to (t, r) (e.g. using the R function `utils::adist`) we obtain:

r:	b	{	s	t	@	l
t:	b	#	s		@	l
edit:	M	S	M	D	M	M

where edit operations are marked M(atch), S(ubstitution), I(sertion) and D(eletion). This result, though not making use of the orthographic form originating both t and r, reflects the fact that it is the A in BASTLE that was produced as `#` instead of `{`, hence S: `{`→`#`, and the T was realised as `t` in r but mute in t, hence D: `t`→. However, the

Levenstein algorithm often produces results that clearly misrepresent the phonemic edit pattern. An example is offered by the nonword ADHORB with reference `{d9b` and target `@dh$b`:

r:	`{`	`d`	`9`		`b`
t:	`@`	`d`	`h`	`$`	`b`
edit:	S	M	S	I	M

where the intuition is that the H was realised as `h` in t but mute in r, hence the insertion should be of `h`, not of `$`, and the substitution should be `9`→`$`, not `9`→`h`. This example shows how easy it is to get spurious errors which (i) pollute the model error list with serious errors that may lead to think that the model is doing really bad, and (ii) mask the real errors.

The solution we devised consists in giving priority to substitution errors between phonemes belonging to the same class. In the example above, `9` to `$` are both vowels (diphthongs), hence they are closer to each other than to `h`, which is a consonant. So it is more likely that a substitution occurs from `9` to `$` rather than from `9` to `h`. The alignment procedure uses a multi-tier class hierarchy. In our implementation we defined two tiers, the first one groups phonemes roughly by place and manner for consonants and by height and frontedness for vowels, while the second, broader one groups vowels and consonants. For example, `p` and `b` belong to the same first tier class (bilabial plosives), and then to the second tier class of consonants. `I`, `i` and `7` belong to the first tier class of front high vowels (diphthongs) and to the second tier class of vowels. The procedure uses the basic Levenshtein alignment algorithm on different class tiers in a recursive way, as explained below.

The core idea is to take a pair of corresponding phoneme strings according to a sequence of proper contiguous edit operations (i.e. all except M) and keep mapping them to a higher level of phonemic classes until either a match (M) appears or we reach the top of the hierarchy. Algorithm 1 is the exact description of the procedure as implemented by function EDITPATTERNS, which takes as input target t, reference r, and the phonemic hierarchy described by the table *classTable*, as well as a pointer to the current level in the hierarchy l, i.e. a column index in *classTable*. The function calls MAP($t, l_1, l_2,$ *classTable*), which returns a string obtained by mapping the symbols in t belonging to column l_1 in *classTable* to the corresponding symbols in column l_2.

Let us use the last example to illustrate function EDITPATTERNS. For simplicity, let us use only one level above phonemes, i.e. C(onsonant) and V(owel). First Levenstein alignment is computed on $t =$ `@dh$b` and $r =$ `{d9b` to obtain $e = SMSIM$. Then, each subsequence pair not including a match, here $t_1 =$ `@`, $r_1 =$ `{` and $t_2 =$`h$`, $r_2 =$ `9`, are first mapped to the upper class level, thus obtaining $t_1 = V$, $r_1 = V$, and $t_2 = CV$, $r_2 = V$, then EDITPATTERNS is recursively applied to each of those pairs. The first pair returns M, which is turned into S, the second returns IM which is turned into IS. These two subsequences are replaced into the initial Levenstein alignment $e = SMSIM$ obtaining $e = SMISM$, i.e.:

Algorithm 1 Phonemic class-aware alignment

function EDITPATTERNS(t, r, l, classTable)
    $e \leftarrow$ LEVENSTEIN(t, r)
    if $l =$ top level of classTable $\vee$ LENGTH$(t) = 1$ **then**
        RETURN(e)
    else
        for all $t' \leftarrow t[i : i+j], r' \leftarrow r[i : i+j]$ such that $M \notin e[i : i+j] \wedge (e[i-1] = M \vee i = 1) \wedge (e[i+j+1] = M \vee i+j = \text{LENGTH}(t))$ **do**
            $t' \leftarrow$ MAP$(t', l, l+1, \text{classTable})$
            $r' \leftarrow$ MAP$(r', l, l+1, \text{classTable})$
            $e' \leftarrow$ EDITPATTERNS$(t', r', l+1, \text{classTable})$
            for all k such that $e'[k] = M$ **do**
                $e'[k] \leftarrow S$
            end for
            $e[i : i+j] \leftarrow e'$
        end for
        RETURN(e)
    end if
end function

r:	`{`	`d`		`9`	`b`
t:	`@`	`d`	`h`	`$`	`b`
edit:	S	M	I	S	M

a more plausible description of the phonemic errors than the one obtained by pure Levenstein alignment.

As a demonstration of the effectiveness of the proposed procedure, the list of different phonemic errors for Sequitur (see section 4) reported around 400 different edit error types when we initially applied Levenstein algorithm, and around 200 when we applied EDITPATTERNS. It is very likely that those extra 200 error types were artifacts. Moreover, the most common error (`9`→`$`) was reported with a frequency of 8% using Levenstein alignment, against more than 9% using EDITPATTERNS, suggesting that the missing 1% was masked by artifacts.

2.2 Mining frequent adjacent phonemic errors

A further issue concerns adjacent phonemic errors that have a common origin, e.g. `S`→`s` followed by →`h` in a nonword containing the SH letter sequence. To identify those sequences of errors we applied the SPADE algorithm as implemented in the R package `arulesSequences`. The main idea is to consider an edit operation as an event of length one, and a sequence of event as a sequence of contiguous edit operations (see Zaki (2001) for formal definitions of event and sequence in this context). For example,

for target `bEshQm` and reference `bESQm`, we have a sequence of length two, (`S`→`s`, →`h`). Sequence rules of the form $e_1, \ldots e_{n-1} \Longrightarrow e_n$, where e_i are single edit operations (or events), are then mined on the entire set of alignments. If a sequence occurs often enough it will be captured by a rule, for example `S`→`s` $\Longrightarrow$ →`h`. When this occurs, instead of reporting the single edit errors separately, we reported it as a whole, i.e. `S`→`sh` (see Cluster error type in section 4). The SPADE algorithm requires to specify a number of parameters, which we tuned empirically (*support* = 0.002, *confidence* = 0.5). Note that in general the quality of the solution depends on the amount of data.

Applying SPADE to the errors of Sequitur identified the following error sequences: `7`→`2@R`, `w`→`ju`, `S`→`sh`, `#`→`8R`, `7`→`@R`, `7`→`2R`, `gh`→`x`. Note that the pattern `S`→`sh`, which we initially identified by inspection, is included in the list. The appearance of many `R`'s flagged an issue related to different transcription conventions in CELEX and in Mousikou, Sadat, Lucas, and Rastle (2017), which we amended (see section 4.1).

3 IPA to DISC conversion table

**IPA**	**DISC**	**IPA**	**DISC**
p	p	ɪ	I
b	b	ɛ	E
t	t	æ	{
d	d	ʌ	V
k	k	ʊ	U
g	g	ɒ	Q
ŋ	N	ə	@
m	m	iː	i
n	n	ɑː	#
l	l	ɔː	$
r	r	uː	u
f	f	ɜː	3
v	v	eɪ	1
θ	T	ɑɪ	2
ð	D	ɔɪ	4
s	s	əʊ	5
z	z	ɑʊ	6
ʃ	S	ɪə	7
ʒ	Z	ɛə	8
j	j	ʊə	9
x	x	c	æ̃
h	h	ɑ̃ː	q
w	w	æ̃ː	0
ʧ	J	ɒː	~
ʤ	_		
n̩	H		
m̩	F		
l̩	P		
*	R		

Table S2
IPA to DISC phonemic conversion table. The first 2 columns show conversions for consonants and the last two columns show conversions for vowels and dipthongs.

4 Patterns of error for CDP++, RC00 and Sequitur

In this section we report on the patterns of pronunciation error of CDP++, RC00 and Sequitur as measured on the 915 nonword stimuli in Mousikou et al. (2017). For Sequitur, we also provide a detailed analysis of some of the most frequent errors, while for CDP++ and RC00 the reader can refer to the rich supplemental material in Mousikou et al. (2017). To determine which edit errors were made we used the automatic methods described in section 2.

The definition we use for error, or mismatch, is the same as in Mousikou et al. (2017), namely a pronunciation that does not match any of those produced by the 41 human subjects in the experiment reported in that article. As a consequence,

whenever such a mismatch is identified, there are several ways to count its edit errors, since in general there is more than one human pronunciation that can be taken as reference. We decided to count errors proportionally to the relative frequency of human references. For example, nonword 'boming' was pronounced `bQmIN` by 24 speakers, `b5mIN` by 11 speakers and `bumIN` by 5 speakers, while one speaker was discarded, so there are 40 realisations in total. Sequitur produced `b@mIN`, which matches none of them. According to those proportions we count $24/40 = 0.6$ `Q` → `@` errors, $11/40 = 0.275$ `5` → `@` errors and $5/40 = 0.125$ `u` → `@` errors. As we noted that several pronunciations produced by only one subject out of 41 were most likely reading errors (e.g. 'rilell' pronounced `rIlfUl`, 'tamcem' pronounced `t{ksim`, 'pispy' pronounced `pIpsi`), we removed singleton pronunciations from the set of references before doing the counting.

Figure S1 shows edit error counts categorised by phonemic class for each model. Insertion and deletion errors are represented as special case of substitution errors where one of the two sides is the empty symbol '-', while Cluster refers to a sequence of adjacent phonemes that belong to the same pattern of error, the latter established by applying sequence mining (see section 2.2). We notice some commonalities among the three models. First, the most frequent edit error is the substitution of a short vowel with another short vowel. Second, the large majority of errors involve only vowels and diphthongs. Third, the most common deletion errors are of plosives, liquids and short vowels. This suggests that the three models, despite profound differences in their assumptions and mechanisms, on a coarse granularity tend to exhibit similar patterns of errors, which are also those that one would expect from a human reader of the English language. At first sight, it seems that what makes Sequitur worse than the others are mainly errors involving vowels and diphthongs.

Tables S3, S4 and S5 report the most frequent edit errors (Count > 5) for CDP++, RC00 and Sequitur, respectively. Note that the Count column reports fractional quantities because of the proportional counting criterion explained above. Columns From and To report the correct phoneme(s) and the corresponding wrong phoneme(s), respectively, where a blank represent the empty symbol for insertions and substitutions. Finally, columns Stimulus, Human pron. and Model pron. report a randomly chosen example for each edit error.

4.1 Sequitur patterns of error in detail

The output from Sequitur was pre-processed in order to eliminate a few obvious discrepancies between the phonemic inventory used in CELEX and in Mousikou et al. (2017). The two most evident discrepancies are:

- in CELEX, syllabic nasals and liquids `C`, `F`, `H` and `P` are used at word end (e.g. 'able' → `1bP`), while in the same context `@N`, `@m`, `@n`, and `@l`, respectively, are used in transcriptions by Mousikou et al. (2017) (e.g. 'vundle' → `vVnd@l`), as well as in the output of CDP++ and RC00;

- in CELEX, syllabic `R` after `@` at word end (e.g. 'clever' → `klEv@R`) is used, while in the same context `@` is used by Mousikou et al. (2017) in transcriptions (e.g. 'flever' → `flEv@`), as well as in CDP++ and RC00.

The results in the main text were obtained after eliminating these discrepancies.

In this section we look at some of the most frequent errors produced by Sequitur. We will limit this case-by-case analysis to the top five errors from Table S5, plus a few others in sparse order.

9 → $. The long vowel `$` never appears either in human transcriptions or in CDP++ and RC00 production in Mousikou et al. (2017). Below a table where a few examples of such error are reported. Column Human modal reports the modal pronunciation from the participants as transcribed by Mousikou et al. (2017), column Similar word and CELEX report a word with a similar orthographic context and its corresponding pronunciation in CELEX.

Nonword	Human modal	Sequitur	Similar word	CELEX
outslaw	6tsl9	6tsl$	outlaw	6tl$
glorak	gl9r{k	gl$r{k	glory	gl$rI
spaulted	sp9ltId	sp$ltId	assaulted	@s$ltId

i → I. This mismatch is frequent in the context of word final 'y', as shown in the table below.

Nonword	Human modal	Sequitur	Similar word	CELEX
darmy	d#mi	d#mI	army	#mI
disty	dIsti	dIstI	misty	mIstI
pifty	pIfti	pIftI	fifty	fIftI

Q → @. This error appears in different contexts, one of them being words starting with 'con'. Note that this error is also frequent for CDP++ (Table S3) and it is the top frequent error for RC00 (Table S4).

Nonword	Human modal	Sequitur	Similar word	CELEX
concume	kQnkum	k@nkjum	consume	k@nsjum
conspact	kQnsp{kt	k@nsp{kt	conspicuous	k@nspIkj9s
contanse	kQnt{ns	k@nt{ns	contend	k@ntEnd

{ → @. This error occurs in different contexts. One is nonwords ending in 'ant' that supposedly are stressed on the 2nd syllable, e.g. 'confant', 'desant'. In CELEX, 13 words have such characteristic, either monosyllabic or 2-syllabic words with stress on the 2nd syllable and ending in 'ant', while 185 are polisyllabic words ending in 'ant' but stressed earlier than the last syllable. The former words are reported with a pronunciation ending in `{nt`, the latter in `@nt`. As Sequitur was trained on a

dictionary that did not contain syllable boundary markers, it is likely to have taken the majority as the winning pattern, hence always `@nt`. RC00 does not do this type of error (it has a concept of syllable). Another pattern are words starting with 'adh'. There are 13 of them in CELEX, all reported with a pronunciation starting with '@dh'.

Nonword	Human modal	Sequitur	Similar word	CELEX
desant	dIs{nt	dIz@nt	recant	rIk{nt
adhace	{dh1s	@dh1s	adhered	@dh7d

E → I. This error occurs regularly in nonwords where the 'es' or 'ex' letter cluster appears.

Nonword	Human modal	Sequitur	Similar word	CELEX
explave	Ekspl1v	Ikspl1v	explain	Ikspl1n
esond	EsQnd	IsQnd	despond	dIspQnd

{ → #. This error can occurs in nonwords where 'a' is the only vowel in the syllable.

Nonword	Human modal	Sequitur	Similar word	CELEX
chansem	J{ns@m	J#ns@m	chance	J#ns
mellaph	mEl{f	mEl#f	telegraph	tElIgr#f
nasple	n{sp@l	n#sp@l	candle	d#ns

→ I. Insertion of `I` occurs in 'ai' context, produced as `1I` instead of `1`. The `1I` sequence never appears in human transcriptions or in RC00 production, while it appears once in CDP++ ('blaicing' → `bl1IN`).

Nonword	Human modal	Sequitur	Similar word	CELEX
blaicing	bl1sIN	bl1IsIN	prosaically	pr5z1Ik@lI
daible	d1b@l	d1Ib@l	dais	d1Is

Unpronounceable output. A few vowel deletions produced unpronounceable output. These cases can occur as Sequitur does not use or derive any explicit phonotactic rule.

Nonword	Sequitur
burdep	b3dp
chootaf	JUtf
deshict	dSIkt
flactuse	fl{kJz
gredest	grdIst
leleg	llEg
mortesh	m9JS
outflign	6tfln
stovem	st5vm
tamcem	t1msm

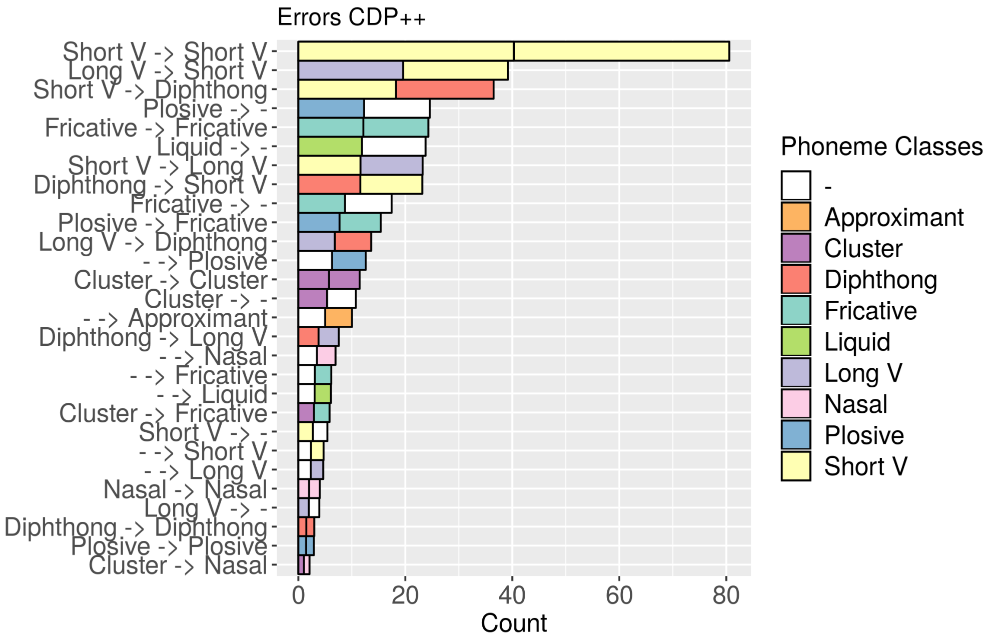

(a) CDP++

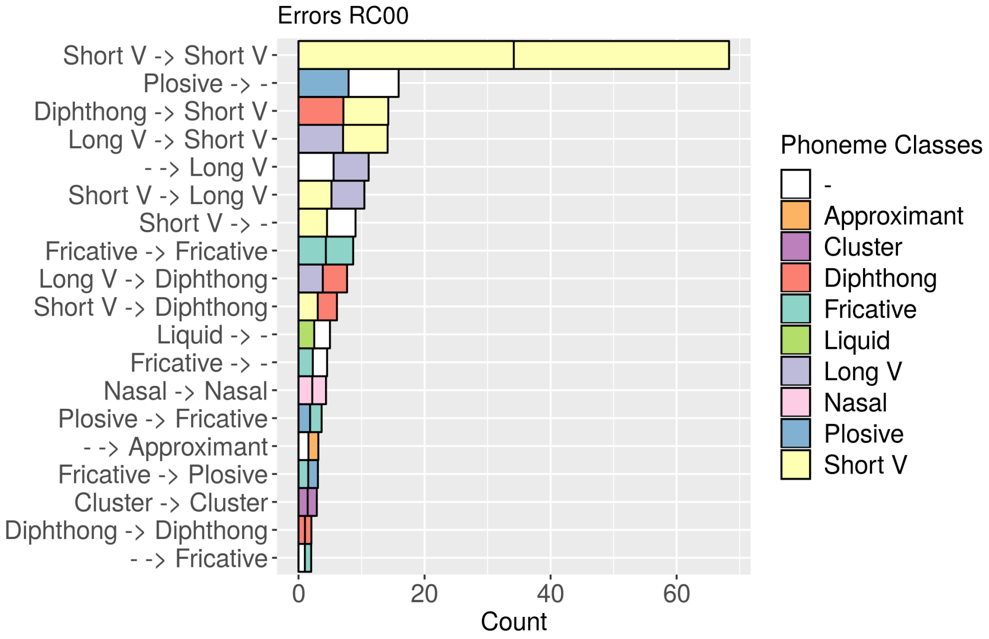

(b) RC00

Figure S1. Edit error counts categorised by phonemic class. ‘V’ stands of Vowel. ‘-’ stands for absent, e.g. ‘Plosive → -’ is a deletion of a plosive and ‘- → Short V’ is an insertion of a short vowel. ‘Cluster’ denotes any sequence of adjacent phonemes, e.g. ‘Fricative → Cluster’ means a substitution of a fricative with more than one phoneme. The three panels report errors for (S1a) CDP++, (S1b) RC00 and (S1c) Sequitur, respectively, up to a count > 2.

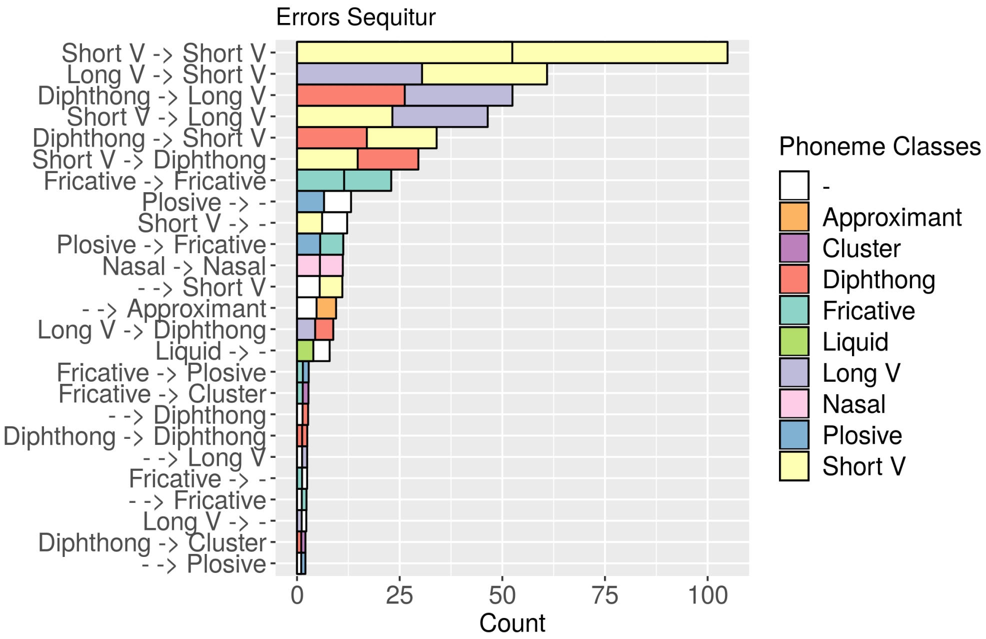

(c) Sequitur

Figure S1. (Continued)

From	To	Count	Proportion	Stimulus	Human pron.	Model pron.
l		23.56	0.05	bartless	b#tl@s	b#t@s
{	@	23.51	0.05	abast	{b{st	@b#st
i	I	22.06	0.05	baicy	b1Si	br1kIN
{	#	18.52	0.04	abast	{b{st	@b#st
s	z	17.16	0.04	asush	{sUS	{zVS
k	s	12.43	0.03	celky	kElkI	sElkI
s		12.38	0.03	aslare	{sl8	@l@
Q	@	11.65	0.03	befon	bIfQn	bEf@n
{	1	11.47	0.03	cervak	s3v{k	s3v1k
Q	5	11.12	0.03	astond	{stQnd	@st5nd
Ul		10.74	0.02	bouseful	b6sfUl	b6sf
E	I	10.61	0.02	bebaft	bEb{ft	bIb#ft
	j	10.02	0.02	concume	kQnkum	k@nkjVm
k		9.92	0.02	baininx	b1nIks	b1nIn
I	2	8.95	0.02	chasime	J{sIm	J1z2m
t		8.09	0.02	bastle	b{st@l	b#s@l
I	E	7.98	0.02	amett	{mIt	#mEt
	d	7.97	0.02	afflove	{flVv	@fluvd
E	@	6.78	0.02	beglets	bEglEts	bIgl@ts
{	I	6.19	0.01	denast	dIn{st	dEnIst
@s	z	5.57	0.01	bafeness	b1fn@s	b{finz
5	u	5.53	0.01	afflove	{fl5v	@fluvd
I	@	5.36	0.01	bartless	b#tlIs	b#t@s
9	@	5.26	0.01	chasorm	J{s9m	J1z@m
i	1	5.01	0.01	arreme	{rim	@r1m

Table S3

Most frequent errors for CDP++.

From	To	Count	Proportion	Stimulus	Human pron.	Model pron.
Q	@	30.37	0.15	cervok	s3vQk	s3v@k
{	@	21.67	0.10	advort	{dv9t	@dv9t
	i	11.13	0.05	bafeness	b1fn@s	b1fin@s
g		7.86	0.04	blingle	blINg@l	blIN@l
9	@	7.36	0.04	forbene	f9bin	f@bin
b		6.80	0.03	kemble	kEmb@l	kEm@l
s	z	6.71	0.03	atuse	{tjus	@tjuz
#	@	6.06	0.03	adnarb	{dn#b	{dn@b
E		5.91	0.03	esede	EsEd	ist

Table S4

Most frequent errors for RC00.

From	To	Count	Proportion	Stimulus	Human pron.	Model pron.
9	$	39.34	0.08	adhorb	{dh9b	@dh$b
i	I	30.77	0.06	baicy	b1si	b1sI
Q	@	22.84	0.05	boming	bQmIN	b@mIN
{	@	21.56	0.05	abast	{b{st	@b#st
E	I	21.22	0.04	befley	bEfli	bIflI
{	#	15.57	0.03	abast	{b{st	@b#st
@	I	11.74	0.02	befrets	b@frEts	bIfrIts
I	2	10.75	0.02	attipe	{tIp	@t2p
s	z	10.50	0.02	adase	{d1s	@d#z
	j	9.51	0.02	buseness	busn@s	bjusnIs
I	i	9.24	0.02	bebaft	bIb{ft	bib#ft
E	i	8.66	0.02	arend	{rEnd	@rind
u	V	7.81	0.02	afflove	{fluv	@flVv
5	@	7.56	0.02	boming	b5mIN	b@mIN
n	N	7.42	0.02	cherinx	JErInk	JErINks
Q	5	7.41	0.02	accoll	{kQl	{k5l
u	U	7.33	0.02	chootaf	Jut{f	JUtf
3	@	6.05	0.01	fipstire	fIpst3	fIpst2@
{	1	5.76	0.01	adhace	{dh{s	@dh1s
	I	5.42	0.01	blaicing	bl1sIN	bl1IsIN
{	$	5.11	0.01	devall	dIv{l	div$l
g	_	5.10	0.01	budgord	bVdg9d	bV_$d

Table S5
Most frequent errors for Sequitur.

5 Microsoft Speech Synthesizer

The Microsoft Speech Synthesizer[1] was used in Experiments 1 and 2. The voice we selected was "en-GB, Hazel", as it has a distinct Southern British English accent (e.g. contrary to the default "en-US Anna"). This voice is not generally pre-installed on Windows 7 [2], the OS that was used in the synthesis phase of our experiments. Stimuli are constructed using the Speech Synthesis Markup Language (SSML)[3], which allows to control speed, insert pauses, and importantly specify a prompt input in terms of phonemic transcription as alternative to orthography. The only missing feature (at the time the experiments were run) was the control on lexical accent position, which can be specified in SSML but was ignored by the synthesiser. The generation of audio files was managed via Windows Power Shell scripting[4].

[1] https://docs.microsoft.com/en-gb/previous-versions/office/developer/speech-technologies/hh361644(v=office.14)

[2]https://superuser.com/questions/590779/how-to-install-more-voices-to-windows-speech,
https://social.technet.microsoft.com/Forums/windows/en-US/c7409091-087e-48f2-8178-bcd918799054/add-tts-voice-to-windows-7-and-set-as-default?forum=w7itproinstall

[3]https://www.w3.org/TR/speech-synthesis11/

[4]https://bugs.chromium.org/p/chromium/issues/detail?id=88072,
https://learn-powershell.net/2013/12/04/give-powershell-a-voice-using-the-speechsynthesizer-class/

Below we reproduce an example SSML prompt script for the phonemic stimulus `{d1n`:

```
<?xml version="1.0"?>
<speak version="1.0" xmlns="http://www.w3.org/2001/10/synthesis" xml:lang="en-GB">
    <voice name="Microsoft␣Server␣Speech␣Text␣to␣Speech␣Voice␣(en-GB,␣Hazel)">
        <prosody rate="slow">
            1 <break strength="weak"/>
        <phoneme alphabet="x-microsoft-ups" ph="␣AE␣D␣E␣+␣I␣N␣">x </phoneme>
        <break strength="weak"/>
    </prosody>
    </voice>
</speak>
```

Note that the phonemic transcription has to be converted into MS UPS phonetic alphabet[5], and that an "x" was used as a placeholder for the orthography, which is ignored when a phonemic transcription is provided.

6 Experiment 1

Table S6 lists the 53 cases in which the median score for the Human Modal pronunciation was "Probably not OK" or worse in Experiment 1. Within such cases, we define "Best pronunciation" as a pronunciation of the same orthographic stimulus whose median score is "Good" or "Very good" (to be on the safe side), and "Best category" as the category that obtained that score. When more than one Best Pronunciation occurred, they are all reported (this occurred only for the nonword COMBIRE).

Table S7 lists the 63 cases in which the median score for the Deliberate Error pronunciation was "Probably OK" or better in Experiment 1. The Human Modal pronunciation is also reported for comparison.

Table S6

Human Modal pronunciations rated as implausible in Experiment 1. Whenever one or more alternative pronunciations of the same orthographic stimulus were rated as plausible (in 33/53 cases), those are indicated in the last two columns.

Orthography	Human Modal	Contains 9	Best category	Best pronunciation
adhorb	{dh9b	yes		
advore	{dv9	yes	Sequitur	{dv$
advort	{dv9t	yes	Sequitur	{dv$t
baininx	b1nINk	no	Minor	b1nIks
baulind	b9lInd	yes		
bebaw	bib9	yes	Sequitur	bib$
beshaw	bIS9	yes		
bourted	b9tId	yes	Sequitur	b$tId
budgord	bVdg9d	yes		
chasorm	J{s9m	yes		
cherinx	JErInk	no	Sequitur	JErINks
combire	kQm2@r	no	Minor	kQmbi@r
combire	kQm2@r	no	Sequitur	k@mb2@

Continued on next page

[5]https://msdn.microsoft.com/en-us/library/hh362879.aspx

Table S6 – continued from previous page

Orthography	Human Modal	Contains 9	Best category	Best pronunciation
condaw	kQnd9	yes		
darmy	d#mi	no		
debort	dIb9t	yes	Sequitur	dib$t
deforn	dIf9n	yes	Sequitur	dIf$n
devort	dIv9t	yes	Sequitur	div$t
distorm	dIst9m	yes	Sequitur	dIst$m
drafforn	dr{f9n	yes	Sequitur	dr@f$n
ejorn	E_9n	yes	Sequitur	I_$n
forbene	f9bin	yes		
forebew	f9rbju	yes	Sequitur	f$bu
forefops	f9fQps	yes	Sequitur	f$fQps
fornoof	f9nuf	yes	Sequitur	f$nuf
forsive	f9sIv	yes		
forsose	f9s5z	yes	Sequitur	f@s5s
fullorn	fUl9n	yes	Sequitur	fUl$n
glorak	gl9r{k	yes	Sequitur	gl$r{k
grauntul	gr9nt@l	yes		
jilnaw	_Iln9	yes	Sequitur	_Iln$
jorteb	_9tEb	yes		
jorteg	_9tEg	yes		
laudul	l9dj@l	yes		
malder	m9ld@	yes	Minor	m5ld@
mortesh	m9tES	yes		
outhort	6th9t	yes	Sequitur	6th$t
outslaw	6tsl9	yes	Sequitur	6tsl$
outsmaw	6tsm9	yes	Sequitur	6tsm$
preforn	prif9n	yes	Sequitur	prif$n
prevork	prIv9k	yes		
probord	pr5b9d	yes	Sequitur	pr@b$d
rorond	r9rQnd	yes		
sheruch	S3VJ	no		
smorak	sm9r{k	yes		
sorglom	s9glQm	yes	Sequitur	s$gl@m
sorknom	s9knQm	yes		
sormand	s9m{nd	yes	Sequitur	s$m@nd
spaulted	sp9ltId	yes	Sequitur	sp$ltId
tactord	t{kt9d	yes	Sequitur	t{kt$d
ulhork	Vlh9k	yes	Sequitur	Vlh$k
umpord	Vmp9d	yes		
ungourn	Vng9n	yes		
unlourt	Vnl9t	yes	Sequitur	Vnl$t

Table S7

Deliberate Error pronunciation rated as plausible in Experiment 1. Human Modal pronunciation reported for comparison.

Orthography	Deliberate Error	Median Response	Human Modal
adase	{d~s	Good	{d1s
adhace	{dt1s	Probably OK	{dh1s
adnarb	{dnHb	Good	{dn#b
allyl	{jIl	Probably OK	{lIl
alyth	3lIT	Good	{lIT
arroste	{r3st	Probably OK	{rQst
attase	{t@s	Good	{t1s
bairtul	b~t@l	Probably OK	b8t@l
bebaft	bIb2ft	Good	bIb{ft
befley	b3fli	Probably OK	bEfli
bethove	bEth0v	Probably OK	bEth5v
bouseful	b6sf5l	Probably OK	b6sfUl
conclise	kQnkj2s	Probably OK	kQnkl2s
concouse	kQnkHs	Probably OK	kQnkus
consads	kQns{dN	Probably OK	kQns{dz
conshede	kQnS@d	Probably OK	kQnSid
conspact	kQnsp{Tt	Probably OK	kQnsp{kt
crovest	krivEst	Good	kr5vEst
danew	d{nj7	Probably OK	d{nju
dasanx	d{s{Nkm	Probably OK	d{s{Nks
debuse	xIbjuz	Good	dIbjuz
dedrast	dIdr5st	Probably OK	dIdr{st
diburse	dID3z	Probably OK	dIb3z
disgranx	dIpgr{Nks	Probably OK	dIsgr{Nks
effove	Ef5R	Probably OK	Ef5v
ejall	I_#l	Probably OK	I_{l
englast	#ngl{st	Good	Engl{st
etrop	Etr5p	Good	EtrQp
evart	IvHt	Good	Iv#t
falkest	fQlk5st	Probably OK	fQlkEst
faseness	f1snqs	Good	f1sn@s
flancing	flHnsIN	Very good	fl#nsIN
folted	f5ltId	Good	fQltId
imbove	Imb~v	Probably OK	Imb5v
imbroths	ImbrQTN	Probably OK	ImbrQTs
lafeless	l3fl@s	Probably OK	l1fl@s
larceful	l#sfUh	Probably OK	l#sfUl
malmer	mqlm@	Probably OK	m{lm@
meabick	jibIk	Probably OK	mibIk
misnop	mIsn3p	Probably OK	mIsnQp
outbost	6tb5st	Probably OK	6tbQst
outplink	FtplINk	Very good	6tplINk
pastald	p{stuld	Very good	p{st{ld
perphise	T@f2z	Probably OK	p@f2z
progese	prcgis	Good	pr5gis

Continued on next page

Table S7 – continued from previous page

Orthography	Deliberate Error	Median Response	Human Modal
prorean	`pr0rin`	Probably OK	`pr5rin`
recebs	`jIsEbz`	Probably OK	`rIsEbz`
sherusc	`SUrusk`	Good	`S3rusk`
slomeful	`sp5mfUl`	Probably OK	`sl5mfUl`
steafle	`stif0l`	Probably OK	`stif@l`
surbept	`s3bEJt`	Probably OK	`s3bEpt`
syrgle	`s3g8l`	Probably OK	`s3g@l`
tarceful	`tIsfUl`	Probably OK	`t#sfUl`
tefless	`hEfl@s`	Probably OK	`tEfl@s`
tettume	`tEtFm`	Probably OK	`tEtum`
tuleg	`tul4g`	Probably OK	`tulEg`
twareful	`tw8fUh`	Probably OK	`tw8fUl`
ubtund	`Vbt~nd`	Probably OK	`VbtVnd`
ucide	`juk2d`	Probably OK	`jus2d`
ungouch	`Vjg6J`	Good	`Vng6J`
ungouds	`1ng6dz`	Good	`Vng6dz`
ungould	`Vng9jd`	Probably OK	`Vng9ld`
ungourt	`Vngit`	Good	`Vng9t`

7 Surprise Index

The *Surprise Index* (SI) (Good, 1956) is a statistic based on information theory that quantifies how unexpected is an outcome of an experiment based on the available outcome probabilities. This index takes into account the probability of the outcome as well as the probability of the alternatives. For example, consider a speaker from Mousikou et al. whose pronunciation of a given nonword is different from all the alternatives, i.e. its estimated probability is 1/41. This outcome is very surprising when all other 40 speakers had pronounced that nonword in the same way ($SI = 5.19$ in this case), while it is less surprising when every single speaker produced their own version ($SI = 0$). Conversely, the most frequent option, e.g. when 40 out of 41 pronounced a nonword in the same way, scores a negative value $SI = -5.32$. In general, a positive SI indicates that a pronunciation is surprising, whereas a negative SI indicates a pronunciation that is unsurprising. Formally, if an experiment has K distinct outcomes each one associated to a probability $p_k, k = 1, \dots K$, the SI for outcome i is defined as:

$$SI(i) = -\log_2(p_i) - H(\{p_k\}) = I(p_i) - H(\{p_k\}) \tag{S.1}$$

where $H(\cdot)$ denotes the entropy function, $\{p_k\}$ is the set of probabilities associated to the K events and $I(\cdot)$ is the information content (in bits). Intuitively, SI of an event is the difference between the information carried by that event and the expected value of information of the whole experiment, i.e. the entropy, so if the event contains more/less information than the average event in that experiment then its SI is positive/negative.

Each pronunciation of each subject can be scored with an SI, so if we look at all the 803 nonwords relevant for this experiment pronounced by all speakers we can obtain a profile of each speaker based on 803 SI scores. Figure S2 shows such profiles in the form of Empirical Cumulative Density Functions (ECDFs), one curve per speaker. The plot shows that most of the pronunciations produced by all speakers are

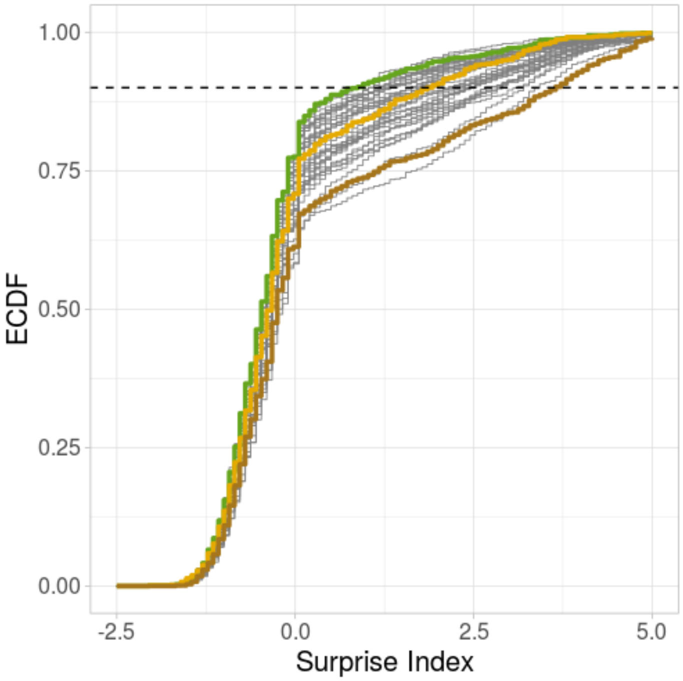

Figure S2. Empirical Cumulative Density Functions (ECDFs) of Surprise Index (SI) defined in Eq. (S.1) and applied to the pronunciations in Mousikou et al.. There are 41 ECDFs, one for each speaker, each one displaying the distribution of SI across all the nonwords pronounced by that speaker. The three thicker curves identify the Modal (leftmost crossing with the horizontal dashed line at 0.9), Typical (mid) and Outlier (rightmost) speakers defined as those speakers whose SI 0.9 quantile is the smallest, median and largest, respectively.

unsurprising (which is expected), as the value $SI = 0$ intercepts cumulative probability values well above the median (0.5 on the vertical axis) for all speakers. However, speakers differentiate themselves roughly in the last quartile of the nonwords. If we intercept the ECDFs at the 0.9 cumulative probability level (dashed line) we get quantiles spanning from 0.8 to 3.6 (consider that the theoretical maximum for this setting is 5.19). We decided to pick the three representative speakers qualitatively defined before by selecting the speakers scoring the minimum, the median and the maximum of the 0.9 SI quantiles. These correspond to the Modal, the Typical and the Outlier Speaker, respectively, and are marked with thicker coloured lines in Figure S2[6].

[6]as the ECDFs cross each other, we cannot use strict domination to determine which one is the

8 Experiment 2 Discrepant Items

From	To	(Corpus, Rating)	(Corpus, -),	(-, Rating)	(-, -)
#	@	3	0	5	1
3	@	1	0	8	0
5	@	10	0	5	0
E	@	24	1	5	4
E	I	78	9	16	8
I	2	19	2	8	6
I	E	38	1	10	4
I	i	16	0	10	3
Q	5	16	2	8	4
Q	@	37	4	25	8
g	_	8	0	5	2
i	I	11	0	24	12
n	N	3	1	6	1
s	z	35	1	15	14
u	U	1	0	6	3
u	V	7	5	5	2
{	#	12	2	15	12
{	1	13	0	6	3
{	@	93	12	29	16

Table S8
Edit errors with highest discrepant cases. The last four columns report how many times edit From → To is found in a stimulus that was judged acceptable by both ratings and corpus-based criteria (Rating, Corp), or not acceptable by both (-, -), or acceptable only by Corpus-based (Corpus, -) or only by ratings (-, Rating) criterion. The selected edit items have at least 5 occurrences of discrepant cases, i.e. the sum of (Corpus, -) and (-, Rating) is ≥ 5*.*

# → @. No synthesis problems, all 5 nonwords acceptable by ratings, 3 acceptable by Phoneticians. Where ratings and Phoneticians in disagreement, median judgements are "Probably ok" and "Probably not ok". No model preponderance.

3 → @. No synthesis problems, all 8 nonwords acceptable by ratings and by Phoneticians. Preponderance of Sequitur (6/8), in 5/8 cases this is the only edit pattern in the pronunciation, with ratings median "Good" or "Very good". The pattern appears almost exclusively in discrepant items.

5 → @. No synthesis problems, all 5 nonwords acceptable by ratings, 2 acceptable by Phoneticians. No model preponderance. The pattern appears in 10 non-discrepant acceptable nonwords.

E → @. Two out of 6 nonwords have synthesis problems, the remaining 4 are all from CDP++ and in co-presence with other edit patterns. This patterns appears in 24 non-discrepant acceptable nonwords and in 4 non-discrepant non-acceptable nonwords.

right/leftmost, so we have to resort to a criterion based on an arbitrary horizontal intercept.

`E` → `I`. Seven out of 25 nonwords have synthesis problems. Of the remaining 18 nonwords, 12 are in agreement with corpus-based criterion. In most of these cases the ratings median was "Probably ok" and "Probably not ok". 12/18 cases are from Sequitur. The pattern is very frequent, with 78 cases in non-discrepant acceptable nonwords and it often appears together with other patterns.

`I` → `2`. No synthesis problems, 5 of the 10 items are from Sequitur, two of which were acceptable by ratings and clearly rejected by Phoneticians. These are nonwords ending in -IND (ETIND, OPPIND) that Sequitur produced with an ending in `2nd`, by analogy with the majority of words in CELEX ending in -IND (e.g. FIND, MIND, BEHIND, etc.). Two other items (THIDFUL as `T2dfUl`, ENBIL as `Enb2l`) were rated "Probably not ok" in Experiment 2 and also rejected by Phoneticians, while acceptable by corpus-based method because of a 1/41 match with a human pronunciation.

`I` → `E`. One of 11 cases had synthesis problems, no model prevalence. In no case it appears in isolation, and it also appears 38 times in non-discrepant acceptable nonwords.

`I` → `i`. No synthesis problems, all 10 cases from Sequitur, all acceptable by ratings, 7 of which acceptable by Phoneticians. All but one item present the edit pattern within a potential prefix BE-, DE-, or RE-, which Sequitur produced with the vowel `i`. Sequitur does not have rules for prefixes, while RC00 and CDP++ do, e.g. RC00 always produces RE- as `rI` when RE- is recognised as proper prefix. Only a minority of human pronunciations for these nonwords were produced with an `i` as first vowel. The rejections in the corpus-based method are consequence of a further mismatch of the second vowel.

`Q` → `5`. No synthesis problems, 5 of the 10 items produced by Sequitur, all accepted by ratings, 3 of the 5 also by Phonetician. Example for the latter are CROLTING produced as `kr5ltIN` probably from analogy with CELEX SCROLL.

`Q` → `@`. One out of 29 items had synthesis problems, the majority of cases (19) are acceptable by ratings and Phoneticians, equally divided between Sequitur and RC00. In several cases nonwords start with CON-, which Sequitur produced as `k@n`. This pattern is very rare in human transcriptions (2%), while it is rather frequent in CELEX (60%). These are 12 cases, 11 of which acceptable by ratings, of which 10 acceptable also by Phoneticians.

`g` → `_`. No synthesis problems, all 5 items acceptable by ratings, of which 3 also by Phoneticians, no model prevalence. Although always mixed with other edit patterns, a case like GEVELD, produced as `_EvElt` by Sequitur and rejected by Phoneticians is likely to be because of `g` → `_`. Sequitur chooses `_` probably in analogy with most (141/158) of the CELEX words starting with GE- and followed by a consonant (e.g. GEL, GENERAL, GERM, GESTURE, etc.).

`i` → `I`. All 24 cases were acceptable by ratings, 13 of which had synthesis problems, all of which from nonwords ending in -Y, so it is likely that final `I` is problematic for this synthesiser. Of the remaining 11 items, 10 are produced by

Sequitur, 9 of which acceptable also by Phoneticians. This edit pattern was already analysed in section 4.1.

n → N. No synthesis problems, 4 of the 7 cases produced by Sequitur. Noticeably, two cases by Sequitur, CONCLISE and CONCLOKE produced with a starting kQN were rated "Very good" both by ratings and by Phoneticians.

s → z. No synthesis problems, no model prevalence, in most cases co-presence of other edit patterns.

u → U. One of 6 cases had synthesis problem (likely due to final I), the remaining 5 cases produced by Sequitur, all acceptable by ratings, 3 of which also by Phoneticians. The two cases rejected by Phoneticians were probably so because of other edit errors (PRULLI as prUl2, TETTUME as tEtjUm). Sequitur produced U in items like DESOOD in analogy with most of the CELEX words ending in -OOD which are related to WOOD or HOOD, these being acceptable by ratings and Phoneticians.

u → V. Four of the 10 items had synthesis problems, 4 of the remaining 6 produced by Sequitur, in 3 of these 4 cases ratings and Phoneticians agreed.

{ → #. No synthesis problems, 12 of the 17 cases by Sequitur, 10 of which acceptable both by ratings and Phonetician. This edit pattern was already analysed in section 4.1.

{ → 1. No synthesis problems, 3 of the 6 cases by Sequitur, all of which accepted by ratings but rejected by Phoneticians. For example APREDS starting with 1, probably in analogy with CELEX APRON and APRICOT.

{ → @. Seven of the 41 cases had synthesis problems, 15 of the remaining 34 produced by Sequitur. This edit pattern is too mixed with others to gain insight, except for the fact that the majority (22/34) of well synthesised items were accepted both by ratings and by Phoneticians.

9 References

Baayen, R. H., Piepenbrock, R., & Gulikers, L. (1995). The CELEX lexical database. linguistic data consortium. *University of Pennsylvania, Philadelphia*.

Bisani, M., & Ney, H. (2008). Joint-sequence models for grapheme-to-phoneme conversion. *Speech communication*, *50*(5), 434–451.

Dempster, A. P., Laird, N. M., & Rubin, D. B. (1977). Maximum likelihood from incomplete data via the EM algorithm. *Journal of the royal statistical society. Series B (methodological)*, 1–38.

Good, I. J. (1956). The surprise index for the multivariate normal distribution. *The Annals of Mathematical Statistics*, *27*(4), 1130–1135.

Levenshtein, V. I. (1966). Binary codes capable of correcting deletions, insertions, and reversals. In *Soviet physics doklady* (Vol. 10, pp. 707–710).

Mousikou, P., Sadat, J., Lucas, R., & Rastle, K. (2017). Moving beyond the monosyllable in models of skilled reading: Mega-study of disyllabic nonword reading. *Journal of Memory and Language*, *93*, 169–192.

Zaki, M. J. (2001). Spade: An efficient algorithm for mining frequent sequences. *Machine learning*, *42*(1-2), 31–60.
